# OmniTFT: an EHR-driven multi-target forecasting model for ICU clinical decision support systems

**DOI:** 10.3389/fdgth.2026.1965956

**Published:** 2026-10-16

**Authors:** Wanzhe Xu, Yutong Dai, Yitao Yang, Zhenhua Zhang, Martin Loza, Weihang Zhang, Yang Cui, Xin Zeng, Sung-Joon Park, Kenta Nakai

**Affiliations:** 1 Department of Computational Biology and Medical Science, The University of Tokyo, Kashiwa, Japan; 2 Human Genome Center, The Institute of Medical Science, The University of Tokyo, Tokyo, Japan; 3 Department of Frontier Research and Development, Kazusa DNA Research Institute, Chiba, Japan

**Keywords:** clinical decision support systems, electronic health records, intensive care units, laboratory results, vital signs

## Abstract

**Background:**

Accurate multivariate time-series forecasting of vital signs and laboratory results is crucial for electronic health record (EHR)–driven clinical decision support systems in intensive care units (ICUs). However, vital signs are susceptible to noise and exhibit rapid fluctuations, while laboratory results are characterized by missing values and measurement lags, making it challenging to forecast heterogeneous ICU variables within a common framework.

**Methods:**

We introduce OmniTFT, a Temporal Fusion Transformer-based multi-target forecasting model for ICU EHR time series. OmniTFT combines state-balanced sliding-window sampling with three training regularizers: frequency-aware embedding shrinkage, historical group-entropy regularization, and shock-alignment regularization. A shared backbone with target-specific heads enables multiple forecasting tasks within a common training and inference framework while preserving target-specific temporal resolutions and forecast horizons. OmniTFT was trained on MIMIC-IV (*n* = 13,277) and independently validated on two cohorts: the MIMIC-III CareVue cohort (*n* = 4,188) and the eICU cohort (*n* = 23,238). Component ablation and sampling sensitivity analyses were conducted using a fixed patient partition and ten different training seeds.

**Results:**

On the MIMIC-IV test set, OmniTFT achieved the lowest MAE for five of eight targets, ranked second for creatinine and respiratory rate, and ranked third for temperature. In a retrospective analysis in eICU, adding the OmniTFT forecast of the subsequent unobserved 24 h to the complete observed record improved discrimination for incident moderate-to-severe acute kidney injury compared with the observed record alone (AUROC 0.763, 95% CI: 0.674–0.852 vs. 0.693, 0.598–0.787, ΔAUROC + 0.070, DeLong *p* = 0.009), and identified 73.3% vs. 57.8% of subsequent events when 30% of patients were flagged for review.

**Conclusions:**

OmniTFT provides a shared-backbone framework for forecasting heterogeneous ICU variables while preserving target-specific temporal configurations. Retrospective results in disease-enriched cohorts support broader external validation and suggest its potential as an EHR-integrated forecasting component for clinical decision support.

## Introduction

Electronic health record (EHR) data are critical to diagnostic decision-making and precision medicine (1). These data originate from distinct sources and exhibit diverse temporal and statistical patterns, reflecting the complexity of clinical practice. In the intensive care unit (ICU), patient conditions recorded in EHRs can change rapidly over short time scales ranging from minutes to hours, posing a challenge for clinicians to accurately predict future physiological states based on clinical judgment (2, 3). This limitation reduces the effectiveness of EHR-driven clinical decision support systems (CDSS) in providing proactive and individualized critical care (4).

In recent years, machine learning methods have been increasingly applied to EHR-based forecasting (5). Their efforts primarily focus on two aspects: (1) Prediction of vital sign trajectories, such as LSTM (5), GRU (6), or traditional regression models (7), which have been investigated in critical-care prediction. Systematic reviews of sepsis prediction and alerting also describe the broader clinical context and variability in evaluation (8, 9). Among recent models, Prophet (10) uses seasonal trends and residual decomposition, while TSMixer (11) employs MLP-based mixing across time steps and feature channels. Their performance on asynchronous ICU measurements depends on preprocessing, available covariates, and the evaluation setting, (2) Prediction of laboratory results. For instance, Nephrocast (12) can learn task-specific relationships and achieves low prediction error in creatinine forecasting. Its extended version, Nephrocast-V (13), further predicts vancomycin trough concentrations 48 h in advance by employing a deep learning model that combines LSTM layers with multi-head attention mechanisms. Although these systems illustrate that predictions based on EHRs can be integrated into clinical workflows, both of them focus on a single laboratory indicator with a short lead time and do not by themselves establish performance across the heterogeneous vital-sign and laboratory tasks evaluated here.

Temporal Fusion Transformer (TFT) combines recurrent temporal encoding, self-attention and variable selection to model heterogeneous covariates (14). This architecture motivates a common forecasting design for ICU targets with different sampling frequencies (7). Feature selection and interpretation are also relevant when many recorded variables may carry overlapping predictive information (15, 16). We evaluate whether additional sampling and regularization mechanisms improve target-specific performance through component ablation. We include frozen pretrained Chronos-2 and TimesFM 3.0 as contemporary forecasting comparators. Both support multivariate prediction without study-specific fitting (17, 18). UniTS supports multiple time-series tasks (19) and is evaluated here through frozen transfer. MTLinear groups related variates using training-data correlations and shares linear forecasting parameters within groups (20). We evaluate its supervised NLinear variant alongside the frozen models.

Here we propose OmniTFT, a deep-learning forecasting framework for ICU EHR data. The framework shares backbone parameters, preprocessing procedures, and a regularized training strategy across targets, while target-specific temporal grids and output heads accommodate differences in temporal resolution and forecast horizon. We evaluate OmniTFT across vital signs, laboratory results, and an oxygenation indicator, and compare it with conventional and task-specific baselines, as well as multi-target forecasting models. On the MIMIC-IV test set, OmniTFT achieved the lowest MAE for 5 of 8 prediction targets and ranked among the top three evaluated models for every target. We further evaluate its generalizability and robustness through external cohort validation, component ablations, sampling sensitivity analyses, and repeated training with multiple random seeds. In a retrospective AKI analysis, adding OmniTFT forecasts of the subsequent unobserved period to the observed record improved risk discrimination for moderate-to-severe AKI. These results support OmniTFT as a common methodological framework for forecasting ICU variables with different temporal characteristics and suggest its potential as an EHR-integrated forecasting component for clinical decision support.

## Methods

### Data source and study population

This study employed three publicly available critical care databases with strictly separated roles in model development and external validation. Only MIMIC-IV was used for model development and training. MIMIC-IV v2.2 (21) was divided by patients into training at a 7:2:1 ratio, and model training was repeated with 10 random seeds. MIMIC-III CareVue and eICU were completely held out from model training and were used exclusively as two independent external validation cohorts.

For the first independent validation cohort, we used the MIMIC-III Clinical Database CareVue subset v1.4 (22) rather than the full MIMIC-III release. This subset was specifically constructed by PhysioNet to avoid patient overlap with MIMIC-IV by retaining CareVue-era ICU stays and excluding individuals represented in MIMIC-IV. We therefore used MIMIC-III CareVue as a patient-non-overlapping temporal external validation cohort. The eICU Collaborative Research Database v2.0 (23) was used as the second independent external validation cohort and provided multicenter evaluation across a distinct hospital population. Neither external cohort participated in model training, fine-tuning, hyperparameter optimization, or model selection.

### Data preprocessing

We included adult ICU admissions satisfying at least one prespecified phenotype definition for sepsis, acute respiratory distress syndrome (ARDS), or acute kidney injury (AKI) (24–26). We initially filtered out non-physiological records (e.g., administrative flags and text-based nursing notes) using the item definition tables provided by each database, retaining vital signs and quantitative laboratory measurements. To harmonize measurements across databases, variables were mapped according to their clinical definitions, database item dictionaries, and measurement units. Measurements representing the same physiological quantity were converted to a common unit and assigned to a common feature definition. When database-specific measurements were not clinically equivalent, they were retained as separate source-specific channels rather than being forcibly merged.

To ensure sufficient target history for window-based forecasting, prediction windows were excluded when more than 30% of the target values were unobserved within the corresponding encoder window after temporal resampling. After temporal resampling, time-varying variables were forward-filled within each ICU admission using only observations available at or before the corresponding grid time, thereby preventing future measurements from entering the model input. Forward filling was applied to model inputs only; forecast errors were computed only at forecast positions with an observed target value. Remaining missing continuous values, including leading missing values and variables unavailable in a given external database, were imputed using the corresponding median estimated from the MIMIC-IV training set. This fixed training-set fallback uses no statistics from the evaluation cohorts and can be applied identically at prediction time, consistent with recommendations for handling missing predictors when validating and applying prediction models (27, 28). Causal forward filling with a fixed fallback is common in ICU time-series pipelines (29), and population median imputation has performed comparably to more complex approaches in clinical prediction models (30). Its potential influence was examined for respiratory-rate forecasting in a dedicated sensitivity analysis (Table 5). Missing categorical variables were represented by an explicit “Unknown” category.

Continuous variables were subsequently standardized using the mean and standard deviation estimated exclusively from the MIMIC-IV training set. The same imputation and scaling parameters were applied unchanged to the internal validation and test sets and to the MIMIC-III and eICU cohorts, no statistics from these evaluation cohorts were used to determine preprocessing parameters (29). Each record was associated with a de-identified patient identifier and a measurement timestamp. After applying exclusion criteria and preprocessing, the final cohorts comprised 13,277 admissions in MIMIC-IV, 4,188 in MIMIC-III, and 23,238 in eICU (Supplementary Table S1). Supplementary Table S2 lists 140 clinical-variable entries, retaining source and unit specific channels separately. Demographic and admission covariates include age, height, weight, gender, race, and admission type. Input variables comprised vital signs, laboratory results, derived indices, and pharmacological variables. Variables absent from an external cohort were treated as missing at inference.

For blood pressure forecasting, the target was invasive mean arterial pressure (MAP, mmHg) obtained from arterial-line measurements. When multiple MAP measurements were available within the same 10 min interval, their median was used to represent that interval. The SF ratio was calculated as SpO_2_/FiO_2_. SpO_2_ and FiO_2_ were first aligned to the 10 min vital-sign grid. When multiple measurements occurred within the same interval, they were aggregated using the same resampling rule as the other vital signs. Because SpO_2_ and FiO_2_ are often recorded asynchronously, FiO_2_ was aligned using the most recent measurement available at or before each 10 min grid point according to the same causal forward-filling procedure used for other time-varying covariates, no future FiO_2_ values were used. FiO_2_ values recorded as percentages were converted to fractions before calculation of the SF ratio. If no valid preceding FiO_2_ measurement was available within the prespecified carry-forward interval, the SF ratio at that time point was treated as missing. We then organized the targets within a multi-target framework using two clinically matched temporal grids: vital signs and the SF ratio were resampled at 10 min intervals, while laboratory results were resampled at 2 h intervals to reflect their sparser collection patterns. Accordingly, the unified model used 12 h histories to predict the subsequent 2 h for vital signs and the SF ratio, and 24 h histories to predict the next 24 h for laboratory results. The model comparison retained the available input and forecast positions after preprocessing (Supplementary Section S3.2). The explanation analyses used the same target-specific temporal grids (Supplementary Section S6). To address the imbalance between relatively stable and highly dynamic physiological periods, we implemented a state-balanced sliding window sampler. Specifically, for each target trajectory, we slid fixed-length windows over the time series, calculated a fluctuation score over the prediction horizon (defined as the difference between the maximum and minimum target values), and labeled each window as volatile if the score exceeded a target-specific threshold, or stable otherwise. During training, windows were sampled to ensure approximately equal representation of stable and volatile states, thereby preventing the model from overfitting to predominantly stable segments. State balancing was applied only during model training. Validation, test and external evaluation retained the naturally occurring distribution of stable and volatile windows. These target-specific thresholds are modeling choices rather than clinical diagnostic criteria: each was fixed before model training to represent a short-horizon change that is clinically meaningful rather than monitor-level noise, and none was tuned on the test set or the external cohorts. Their sensitivity is evaluated in Table 4, and all threshold values are listed in Supplementary Section S2.1.

### Vanilla TFT

We describe the Vanilla Temporal Fusion Transformer (TFT) for comparison. The original Temporal Fusion Transformer uses a sequence encoder to model local temporal patterns and a self-attention mechanism to capture long-range dependencies. It encodes both static and time-varying covariates and employs a variable selection network and a gating mechanism to regulate information flow. It supports multi-temporal predictions and produces probabilistic outputs via a quantile loss (14).

### OmniTFT

OmniTFT extends the Temporal Fusion Transformer for multi-target forecasting from ICU EHR data through a shared backbone with target-specific output heads. All targets use the same backbone parameters and common EHR representation, while each target retains its own temporal grid and output head to accommodate differences in target definition and forecasting horizon.

During training, mini-batches are constructed and processed according to the temporal grid of the corresponding target. Each target-specific batch updates the shared backbone together with its corresponding output head, whereas the other target heads remain unchanged. Consequently, the shared backbone is optimized using supervision from all forecasting targets, while the target-specific heads preserve task-specific output structures.

During inference, forecasts for different targets are generated through separate forward passes, because their temporal grids and output definitions differ. Each pass uses the corresponding target-specific grid and output head while reusing the same trained backbone parameters. Thus, “unified” refers to the shared model parameterization and common preprocessing, training, and inference framework across targets, rather than to simultaneous generation of all target forecasts within a single forward pass.

OmniTFT is trained using the TFT quantile prediction loss together with regularization terms applied to categorical embeddings, historical variable-selection weights, and decoder attention. The mathematical specification below defines these components.

### Frequency aware embedding shrinkage

At the embedding layer, long-tailed categorical covariates can inject high-variance directions because each category is assigned a free embedding row even when evidence for that category is scarce. Consider a categorical feature *i* with vocabulary size $V_{i}$ and embedding matrix $W_{i}$. Let $c_{i,k}$ denote the mini-batch count of category *k*. For categories observed in the current mini-batch, we define the empirical batch frequency $p_{i,k}$ and the corresponding row-wise shrinkage term as follows:

$$\begin{array}{c} R_{\mathrm{embed}}^{\left(i,k\right)}=\frac{∥W_{i}[k,:]{∥}_{2}^{2}}{\sqrt{\;p_{i,k}+\varepsilon }},\;p_{i,k}=\frac{c_{i,k}}{\sum_{u=1}^{V_{i}}\;c_{i,u}} \end{array}$$ (1)

Equation 1 defines a frequency-adaptive L2 penalty applied to each embedding row. Because the penalty is inversely weighted by the square root of the empirical batch frequency, rarer categories undergo stronger shrinkage, while more frequent categories are regularized more mildly. Categories absent from the current mini batch are not evaluated during that update step. In the overall embedding regularization, these row-wise terms are first averaged within each categorical feature and then across all categorical features, to specify the relative scaling of the regularizer across vocabularies.

### Hierarchical variable selection

After the categorical embeddings are stabilized, the next source of instability is attention dilution. We therefore compress the original channel-level selection weights into three TFT-native semantic groups: unknown, known, and observed. Let $w_{t}^{hs}$ denote the historical variable-selection weights at time *t* and let *G* be a fixed binary assignment matrix that maps channels to these three groups. The grouped scores and their normalized distributions are defined as follows (Equation 2):

$$\begin{array}{c} s_{t}^{hs}=Gw_{t}^{hs}\in R^{3},\;p_{g}^{hs}(t)=\frac{s_{t,g}^{hs}}{\sum_{g^{\prime }=1}^{3}\;s_{t,g^{\prime }}^{hs}+\varepsilon } \end{array}$$ (2)

Here, ε is a small constant introduced for numerical stability when the summed group weights approach zero. The first relation aggregates channel-level weights into three nonnegative group-level scores, while the second normalizes these scores by their total weight plus ε. Because ε is added to the denominator, the normalized scores sum to slightly less than one rather than lying exactly on the probability simplex. This normalization therefore makes the regularization primarily reflect the relative allocation of weight across semantic groups rather than the overall magnitude of the underlying channel weights. Because only future-known exogenous variables are structurally available on the decoder side, this regularization was applied only to the grouped historical weights. We therefore defined the group regularization as a time-averaged entropy-based concentration penalty on the normalized group scores in $p_{g}^{hs}(t)$:

$$\begin{array}{c} C_{\mathrm{group}}=\frac{1}{E}\sum_{t=1}^{E}H(\;p_{t}^{hs}),H(p)=-\sum_{g=1}^{3}\;p_{g}\log\;p_{g} \end{array}$$ (3)

Minimizing Equation 3 encourages the historical group-selection weights to become more concentrated within each encoder position (31). The original channel-level selection pathways remain available. The entropy penalty acts within each encoder position, temporal-attention stability is evaluated separately.

### Shock- alignment regularization

To encourage alignment between decoder attention and changes in the learned representation, let $A^{\left(m\right)}(t,\tau )$ be the causal self-attention weight from the target time *t* to past time τ≤t for head *m*. We average the attention heads to define a common attention surface (Equation 4):

$$\begin{array}{c} \overset{\text{-}}{A}\;(t,\tau )=\frac{1}{M}\sum_{m=1}^{M}A^{\left(m\right)}(t,\tau ) \end{array}$$ (4)

We summarize recent-context attention by summing causal off-diagonal weights within a window of width W (Equation 5):

$$\begin{array}{c} a_{t}=\sum_{k=1}^{W}\overset{\text{-}}{A}\;(t,t-k) \end{array}$$ (5)

We set W=3 to define a short local context. Under the target-specific resampling grids, this corresponds to a 30 min recent-context window for vital signs and SF ratio, and a 6 h recent-context window for laboratory targets. We then aggregate the attention of temporally adjacent positions to obtain the short-term lookback strength, to avoid trivial terms, self-loops of *t* pointing to *t* are excluded. This window is a modeling choice and need not match the duration of a clinical shock episode. This geometric projection converts a surface into a time series that can be aligned with a state variability index. The companion signal to which this local mass should respond is a measure of the instantaneous variability of the decoder side representation. Let $v_{t}\in R^{H}$ denote that representation. A label-free proxy for representation change is the Euclidean first difference of the representation, defined as $s_{t}^{\left(1\right)}=∥v_{t}-v_{t-1}{∥}_{2}$ where $v_{t}$ means the decoder hidden state at time *t*. First-order differencing summarizes successive latent-state changes through $s_{t}^{\left(1\right)}$. This proxy avoids explicit change point labels, integrates cleanly with end-to-end training, and matches the temporal resolution of the local historical-attention mass.

$$\begin{array}{c} {\tilde{s}}_{t}^{\left(1\right)}=\frac{s_{t}^{\left(1\right)}-\mu_{s}}{\sigma_{s}},{\tilde{a}}_{t}=\frac{a_{t}-\mu_{a}}{\sigma_{a}} \end{array}$$ (6)

Since $a_{t}$ and $s_{t}^{\left(1\right)}$ have different scales and units, naive alignment would be dominated by magnitude instead of temporal co-variation. We apply per-sequence standardization to both (Equation 6). This places both series on a comparable, unit-free scale and ensures that the subsequent loss penalizes discrepancies in relative dynamics rather than absolute size.

For feature types of OmniTFT, covariates were partitioned identically into static, observed-historical, and future-known groups. Static covariates comprised age, height, weight, race, gender, and admission type. Observed-historical covariates comprised all resampled clinical variables available up to the forecast start, including the pharmacological variables described above. No future laboratory or vital-sign values were treated as known covariates.

### Training process

All OmniTFT training and inference runs were executed on a single NVIDIA H100 Tensor Core GPU (SXM5, 80 GB, Hopper architecture, compute capability 9.0) per run. Sixteen H100 GPUs were available to execute independent experimental runs in parallel, no distributed multi-GPU training was used. Within each OmniTFT training run, all forecasting targets share the same backbone parameters. Target-specific mini-batches update the shared backbone together with the output head corresponding to that target, while the other target-specific heads remain unchanged. After training, target-specific checkpoints were saved for inference and deployment, each containing the trained shared backbone together with the corresponding target-specific output head and configuration. During inference, each target is evaluated through a separate forward pass using its corresponding temporal grid and output head while reusing the same trained backbone parameters. The same single-GPU execution scheme was used for the full model, component-ablation experiments, and sampling-sensitivity analyses. The final configuration uses a model width of 128 units, a two-layer LSTM encoder and decoder, 6 attention heads, and a dropout rate of 0.3. Training optimizes with Adam, using a learning rate of 1 × 10⁻^5^, a mini-batch size of 64, gradient clipping at 1.0, and a maximum of 300 epochs. Early stopping is employed with a patience of 10. Hyperparameter optimization was performed via a grid search across seven groups in MIMIC-IV only. Neither MIMIC-III CareVue nor eICU was used for training, fine-tuning, hyperparameter selection, or model selection. Target-specific settings and available explanation configurations are detailed in Supplementary Sections S2.6 and S6. A complete OmniTFT training run required approximately 4–6 h per seed. Each target-specific deployment checkpoint contained approximately 1.83–1.85 million parameters, including the shared backbone and the corresponding target-specific output head. Full settings, parameter counts and memory measurements appear in Supplementary Section S8.

### Ablation and sampling sensitivity

We evaluated one-component ablations of state-balanced sampling, embedding regularization, historical group-entropy regularization, and shock-alignment regularization. For regularization ablations, the corresponding loss term was disabled while the remaining model architecture was retained, removing state-balanced sampling restored natural sampling. The Full configuration and all ablation variants used the same fixed patient partition, preprocessing pipeline, evaluation procedure, and ten matched training seeds. Performance was summarized as mean MAE ± SD across seeds.

Sensitivity analyses varied the target-specific fluctuation threshold to 0.5 × and 1.5 × its reference value while retaining 50% volatile-window sampling, and varied the proportion of volatile windows sampled to 25% and 75% while retaining the reference threshold. Natural sampling, in which stable and volatile windows were sampled according to their original frequencies in the training data without rebalancing, was included as an additional comparison. The target-specific fluctuation thresholds were used only to characterize within-horizon target variability for sampling and did not represent clinical diagnostic criteria. Within each target, variant-minus-Full MAE differences were compared across the ten matched seeds using two-sided paired *t* tests. Benjamini–Hochberg correction (32) was applied separately to the ablation and sampling-sensitivity comparison families. Non-rejection was not interpreted as evidence of equivalence. Training seeds and the paired-testing procedure are described in Supplementary Section S4, target-specific thresholds are listed in Supplementary Section S2.1, and BH-adjusted significance levels are reported in Tables 3, 4.

### Forecasting comparators

We compared OmniTFT with Vanilla TFT, LSTM, GRU, Prophet, Nephrocast, TSMixer, Chronos-2, TimesFM 3.0, UniTS, and MTLinear. For trainable conventional baselines, models were fitted for the corresponding forecasting targets using MIMIC-IV development data and, where supported by the model class, the same preprocessed covariates, target-specific temporal grids, history lengths, and forecast horizons used for OmniTFT. Vanilla TFT retained the underlying TFT architecture without the OmniTFT-specific sampling and regularization components. LSTM and GRU served as recurrent sequence-model baselines, Prophet as a statistical forecasting baseline, TSMixer as an MLP-based multivariate forecasting model, and Nephrocast was evaluated for creatinine forecasting according to its target-specific formulation. To broaden the comparison beyond these conventional and target-specific baselines, we additionally included contemporary multivariate and multi-task forecasting models. Chronos-2 and TimesFM 3.0 were evaluated as pretrained multivariate forecasting models using frozen official weights without study-specific fitting. UniTS was included as a multi-task time-series model and evaluated using a frozen reproduction checkpoint without ICU-specific fitting or tuning. MTLinear was included as a supervised parameter-sharing baseline and used its NLinear formulation fitted on the corresponding MIMIC-IV training and validation partitions. Model-specific architectures, input representations, adapters, fitting procedures, and hyperparameter settings are provided in Supplementary Section S3. Patient-level paired MAE differences between OmniTFT and each contemporary comparator were tested with two-sided paired *t* tests and Benjamini–Hochberg correction (32) (Supplementary Section S3.2, Table S4).

### Explanation stability and subgroup analysis

The explanation analyses used the fixed target-specific deployment checkpoints, frozen preprocessing parameters and one eligible history window per patient. The SHAP summaries were computed at the event level for the seed-2025 model on the full validation set. Grouped permutation SHAP (33) was used to attribute the first-step P50 forecast to the complete historical trajectory of each clinical input. Numerical and reference sensitivity were assessed across repeated algorithm seeds and background samples, and sampling stability was evaluated using 2,000 patient-bootstrap draws (34).

Temporal-attention profiles were derived from the same target-specific patient samples by averaging attention across heads and normalizing over historical positions. Subgroup analyses compared sex, admission age (<65 vs. ≥65 years) (35), and observed-input coverage. These analyses were designed to characterize the numerical, sampling, and subgroup stability of fitted-model explanations rather than to establish causal or physiological mechanisms (36). Full sample definitions, feature-selection criteria, target-specific time settings, and analysis procedures are provided in Supplementary Section S6.

### Sensitivity to external input availability

To evaluate sensitivity to clinical variables unavailable in external datasets, we performed an input-availability analysis using a frozen respiratory-rate model with a 12 h history, a 2 h forecast horizon, and a 10 min temporal grid. The analysis included 174 patients and 1,538 observed future labels. Externally unavailable clinical values were either replaced using reference-partition (the MIMIC-IV training set) medians or means or removed at the source-event level before temporal aggregation. Additional conditions removed the most frequent one or three reference-partition clinical inputs.

Across all conditions, target history, forecast origins, static covariates, and outcome labels were held fixed. Performance was evaluated using observed-label MAE, with uncertainty estimated from 2,000 shared patient-bootstrap draws. Table 5 reports the evaluated conditions, and full implementation details are provided in Supplementary Section S5.

### Retrospective AKI landmark analysis

In the eICU cohort, we fixed one decision time *t* at 24 h after ICU admission, identical for all prediction settings. Every predictor came from data recorded at or before *t*. Eligible stays were those in which adults were still in the unit at *t*, with at least one serum creatinine before *t* and one within the outcome window, stays already meeting stage ≥1 criteria at or before *t* were excluded, leaving only incident events. Baseline creatinine was the nadir of all values up to *t*. The outcome was incident AKI in the 24 h after *t* by the creatinine criteria of KDIGO (26): stage ≥1 (≥0.3 mg/dL above the 48 h nadir, or ≥1.5 × baseline) and stage ≥2 (≥2 × baseline). The primary analysis included 4,685 eligible stays, with 45 incident stage ≥2 events.

Three prediction settings shared the same stays and labels: Real-12 h (creatinine from 12 to 24 h), Real-24 h (all creatinine ≤ *t*), and OmniTFT (Real-24 h plus summaries of the OmniTFT forecast for 24–48 h). Forecasts used twelve encoder steps ending at *t* on the model's native 2-h grid.

For each prediction setting, model selection was performed independently (37) over the same search space of five logistic-regression and twelve gradient-boosting configurations using three inner and five outer hospital-grouped folds. No imbalance correction was applied, and all estimates are at the natural event rate. Given that only 45 stage ≥2 events occurred among 4,685 eligible stays, we used 2,000 paired hospital-cluster bootstrap resamples to quantify uncertainty (34). AUROC 95% CIs for individual prediction settings and pairwise AUROC *P* values were additionally obtained with DeLong's method, and pairwise AUROC differences are shown with 2,000 stay-level bootstrap intervals. We also reported AUROC, non-interpolated average precision (AP) (38), Brier score, calibration intercept and slope, and decision-curve analyses (39) at thresholds of 0.5%, 1%, 2%, and 3%. Secondary analyses included stage ≥1 endpoint and a comparison according to creatinine-measurement density (Supplementary Section S7).

## Results

### Overview of OmniTFT

To prepare inputs for OmniTFT, we selected a dataset for training and internal validation, along with two external validation cohorts (Figure 1A). To distinguish relatively stable and volatile windows, we compute a fluctuation score over the prediction horizon for each sliding window (max minus min of the target within the horizon) and assign a binary state label: windows with scores exceeding a prespecified target-specific modeling threshold are labeled as volatile, and the rest as stable. These labels then guide a sampler that balances stable and volatile windows during training (Figure 1B).

**Figure 1:**
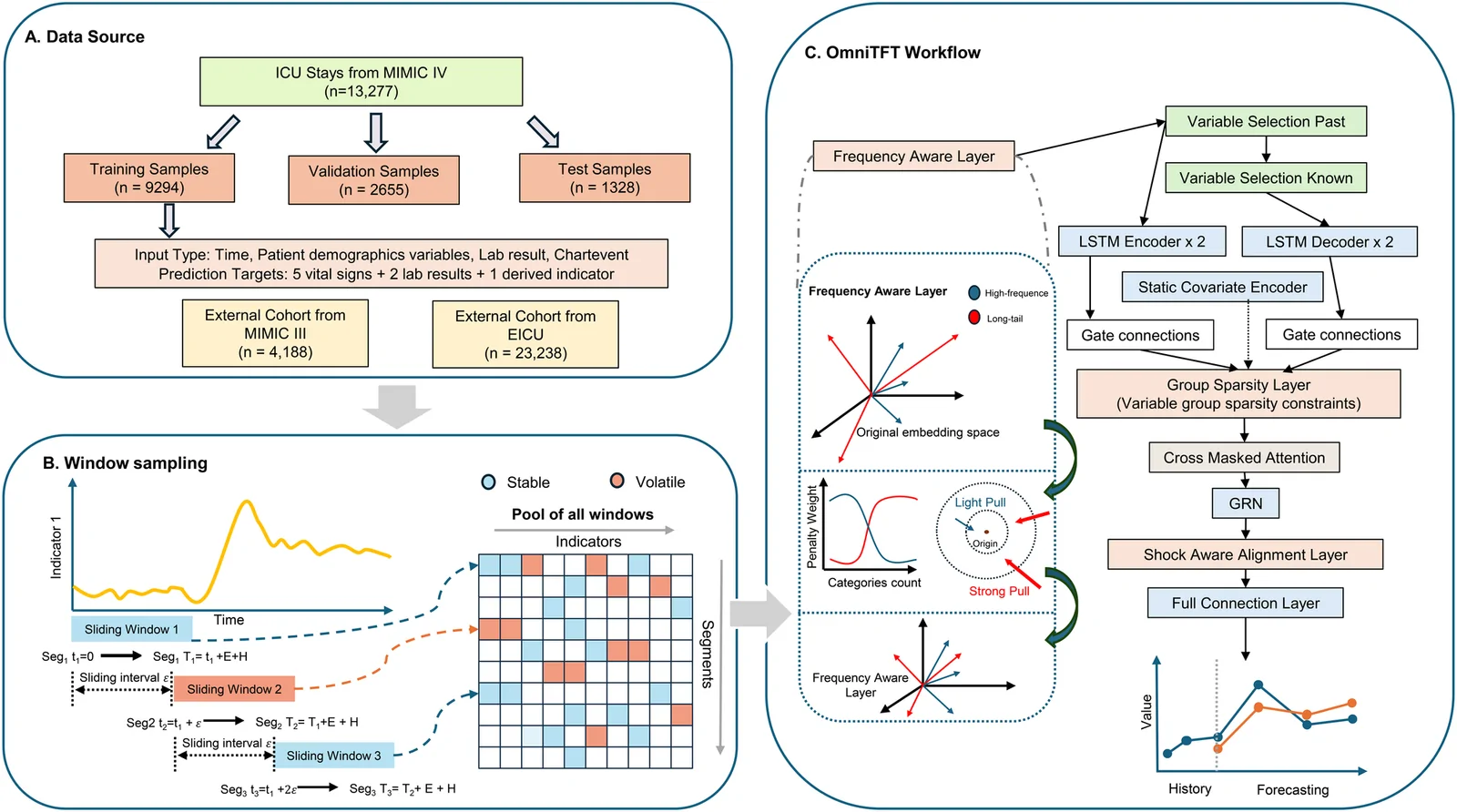
Overview of OmniTFT. **(A)** Study cohort and data split. Eligible MIMIC-IV ICU admissions (*n* = 13,277) were divided into training, validation, and test sets, with MIMIC-III CareVue and eICU used for external evaluation, targets included five vital signs, two laboratory measures, and one derived indicator (SF ratio). **(B)** Window sampling. Sliding encoder–forecast windows were sampled from longitudinal trajectories using a balanced strategy across stable and volatile states. **(C)** OmniTFT workflow. Past, future-known, and static inputs were processed through dedicated modules with frequency-aware embeddings, LSTM encoders/decoders, hierarchical variable selection, attention, gated residual networks, and shock-alignment regularization before final prediction. The backbone parameters are shared across targets, while target-specific temporal grids and output heads are used during training and inference.

Past selected inputs pass through a two-layer LSTM encoder, whose final states initialize the two-layer LSTM decoder for known-future inputs. Static covariates condition variable selection, the encoder's initial states and static enrichment. Gated residual processing and causal self-attention represent temporal dependencies, shock-alignment regularization relates local attention to changes in the learned latent representation (Figure 1C).

For each target, OmniTFT produces forecasts together with variable-selection, temporal-attention, and attribution summaries. Additional targets can be incorporated within the same shared-backbone framework by adding a target-specific temporal grid and output head while retaining the shared backbone parameters.

### Validation of model performance

To evaluate predictive accuracy and cross-cohort performance, we benchmarked five vital signs (blood pressure, heart rate, SpO2, Respiratory rate, and Temperature) and two laboratory results (Creatinine and Lactate) across three ICU datasets: MIMIC-IV's internal test set (21), with MIMIC-III (22) and eICU (23) as external validation sets. We also included the SF ratio, calculated as SpO2 divided by FiO_2_ using bedside pulse oximetry and the recorded fraction of inspired oxygen. We included the SF ratio as a noninvasive oxygenation indicator, in moderate or severe ARDS, prior work reported its association with PaO_2_/FiO_2_ and evaluated risk stratification using the SF ratio together with PEEP after 24 h (40). Table 1 summarizes forecasting errors for all selected targets across the three datasets. Each cell provides paired values for mean absolute error (MAE) and mean absolute percentage error (MAPE) (41). Supplementary Table S3 provides performance summaries across ten training seeds and quantile coverage information for all prediction targets.

**Table 1:** Summary of MAE (MAPE%) across internal and external validation sets.

Label	MIMIC-IV test	MIMIC-III	eICU
Blood Pressure	5.05 (6.67)	6.47 (8.60)	4.98 (6.54)
Heart Rate	4.12 (4.68)	6.29 (7.10)	6.65 (7.02)
Creatinine	0.30 (15.86)	0.42 (22.83)	0.34 (17.09)
SpO2	1.04 (1.09)	1.90 (2.03)	2.15 (2.33)
SF ratio	16.53 (5.24)	20.23 (6.85)	22.25 (6.63)
Lactate	0.35 (18.20)	0.76 (26.03)	0.61 (21.45)
Respiratory Rate	1.72 (8.61)	2.75 (13.82)	2.28 (11.52)
Temperature	0.62 (1.67)	0.55 (1.37)	0.75 (2.08)

MAE, mean absolute error; MAPE, mean absolute percentage error (%).

BP denotes invasive mean arterial pressure (mmHg).

Forecasting errors varied across targets and cohorts (Table 1). Absolute errors increased in external validation for heart rate and the SF ratio, whereas temperature MAE remained below 1 °C in all three cohorts. Relative errors for vital signs were generally in the single-digit percentage range, except for respiratory rate in the two external cohorts. Creatinine and lactate had MAPE values of 15.86%–22.83% and 18.20%–26.03%, respectively. SF-ratio MAE ranged from 16.53 to 22.25, with MAPE of 5.24%–6.85%. Overall, these results indicate cross-cohort forecasting performance within the disease-enriched populations studied, with accuracy varying by target.

### Comparison of OmniTFT with other methods

Table 2 summarizes the performance of OmniTFT and all comparator models under the same evaluation protocol. OmniTFT achieved the lowest MAE for blood pressure, heart rate, SpO_2_, lactate, and the oxygenation target. It ranked second for creatinine (0.30 vs. 0.16 for Nephrocast) and respiratory rate (1.72 vs. 1.23 for TSMixer), and third for temperature (0.62 vs. 0.57 for TimesFM 3.0 and 0.59 for Chronos-2). In patient-level paired comparisons with the four contemporary comparators, OmniTFT had significantly lower MAE in 29 of 32 contrasts after Benjamini–Hochberg correction, the exceptions were temperature (Chronos-2 and TimesFM 3.0 lower) and creatinine vs. Chronos-2 (not significant) (Supplementary Table S4).

**Table 2:** Comparison of OmniTFT with other methods.

Method	BP	HR	SpO_2_	RR	Temp	Creat.	Lactate	SF RATIO
Vanilla TFT	9.48	8.33	2.25	3.08	0.78	1.53	0.99	24.10
LSTM	9.57	9.45	2.16	4.36	0.91	0.63	0.67	24.66
Prophet	10.53	11.37	2.14	4.63	0.77	1.17	1.19	31.12
GRU	9.59	9.47	2.16	4.36	0.81	0.64	0.66	23.51
Nephrocast	—	—	—	—	—	**0** **.** **16**	—	—
TSMixer	6.72	7.39	2.45	**1**.**23**	0.70	0.51	0.88	23.05
Chronos-2	5.62	8.80	1.78	3.98	0.59	0.35	0.85	26.46
TimesFM 3.0	5.30	8.45	1.66	3.77	0.57	0.42	0.92	23.57
UniTS	6.50	9.89	1.88	4.34	0.74	0.39	1.33	56.97
MTLinear	5.81	8.32	1.68	3.73	0.67	0.58	0.72	27.72
OmniTFT (Ours)	**5**.**05**	**4**.**12**	**1**.**04**	1.72	0.62	0.30	**0**.**35**	**16**.**53**

“—” indicates not evaluated or not reported. OmniTFT achieved the lowest MAE for five of eight targets, ranked second for creatinine and respiratory rate, and ranked third for temperature. The comparator models were evaluated under consistent validation procedures, however, most were evaluated on a per-target basis because they do not support OmniTFT's shared-backbone multi-target training across heterogeneous target-specific temporal grids and output heads. Bold values indicate the lowest MAE for each prediction target.

### Ablation study

Component removal increased mean MAE for every target, 29/32 contrasts met within-target BH *q* < 0.05 (Table 3). Removing state balancing increased MAE by 36.92%–60.42% for BP, HR, SpO_2_, and RR, whereas removing group regularization produced the largest MAE increases for the remaining targets. The effects of removing embedding regularization for BP and temperature and shock-alignment regularization for temperature were not statistically significant.

**Table 3:** Ablation of the proposed training components.

Configuration	BP	HR	SpO_2_	RR	Temp	Creat	Lactate	SF RATIO
Full OmniTFT	5.054 ± 0.347	4.119 ± 0.112	1.041 ± 0.020	1.720 ± 0.025	0.619 ± 0.136	0.301 ± 0.016	0.346 ± 0.034	16.529 ± 0.460
w/o state-balanced sampling	6.92 ± 0.36***	6.21 ± 0.33***	1.67 ± 0.10***	2.68 ± 0.18***	0.78 ± 0.05**	0.37 ± 0.03***	0.43 ± 0.03***	18.92 ± 0.51***
w/o embedding regularization	5.31 ± 0.26	4.38 ± 0.20**	1.12 ± 0.07**	1.86 ± 0.12**	0.66 ± 0.04	0.33 ± 0.02***	0.38 ± 0.02*	17.21 ± 0.34**
w/o group regularization	6.14 ± 0.32***	5.52 ± 0.29***	1.58 ± 0.09***	2.41 ± 0.16***	0.91 ± 0.06**	0.41 ± 0.03***	0.48 ± 0.04***	19.87 ± 0.55***
w/o shock-alignment regularization	5.62 ± 0.28***	6.04 ± 0.31***	1.18 ± 0.08**	2.23 ± 0.15***	0.70 ± 0.04	0.34 ± 0.03**	0.39 ± 0.03**	18.34 ± 0.47***
Vanilla TFT	9.48	8.33	2.25	3.08	0.78	1.53	0.99	24.10

MIMIC-IV MAE ± SD across ten matched training seeds, lower is better. Each removal was independently trained, disabling the corresponding sampling rule or loss. Full OmniTFT values are the ten-seed results in Supplementary Table S3. Two-sided paired *t* tests (9 degrees of freedom), with BH correction across four removals per target.

Unstarred tested cells have *q* ≥ 0.05. Vanilla TFT shows its original aggregate mean and was not included in these paired tests.

* *q* < 0.05.

** *q* < 0.01.

*** *q* < 0.001.

### Threshold and sampling sensitivity

Within the tested grid, the reference setting had the lowest mean MAE for all targets (Table 4). Threshold changes increased MAE by 1.76%–6.98%, altered volatile-window sampling increased it by 2.99%–13.95%, and natural sampling by 14.47%–60.42%. Of 40 contrasts, 22 met within-target *q* < 0.05.

**Table 4:** Threshold and state-sampling sensitivity across ten paired training seeds.

Target	S0: Full	S1: 0.5×	S2: 1.5×	S3: 25%	S4: 75%	S5: natural
BP	5.054 ± 0.347	5.18 ± 0.25	5.25 ± 0.26	5.45 ± 0.28*	5.28 ± 0.26	6.92 ± 0.36***
HR	4.119 ± 0.112	4.22 ± 0.19	4.35 ± 0.20**	4.65 ± 0.23***	4.40 ± 0.21**	6.21 ± 0.33***
SpO_2_	1.041 ± 0.020	1.07 ± 0.06	1.10 ± 0.07*	1.18 ± 0.08***	1.12 ± 0.07**	1.67 ± 0.10***
RR	1.720 ± 0.025	1.78 ± 0.12	1.84 ± 0.13*	1.96 ± 0.14**	1.86 ± 0.13**	2.68 ± 0.18***
Temperature	0.619 ± 0.136	0.63 ± 0.03	0.65 ± 0.04	0.67 ± 0.04	0.65 ± 0.04	0.78 ± 0.05**
Creatinine	0.301 ± 0.016	0.31 ± 0.02	0.31 ± 0.02	0.32 ± 0.03	0.31 ± 0.02	0.37 ± 0.03***
Lactate	0.346 ± 0.034	0.36 ± 0.02	0.36 ± 0.02	0.37 ± 0.03	0.36 ± 0.02	0.43 ± 0.03***
SF RATIO	16.529 ± 0.460	16.82 ± 0.29*	17.05 ± 0.31*	17.45 ± 0.34***	17.10 ± 0.32*	18.92 ± 0.51***

Entries are MAE ± SD across ten seeds. Paired *t* tests with BH adjustment across S1–S5 per target.

Unstarred tests have *q* ≥ 0.05. Full/S0 and natural sampling are shared with Table 3.

* *q* < 0.05.

** *q* < 0.01.

*** *q* < 0.001.

S0, reference threshold and 50% volatile-window sampling; S1/S2, threshold × 0.5/ × 1.5, S3/S4, volatile sampling 25%/75%; S5, natural sampling.

### Sensitivity to external input availability

As a targeted sensitivity analysis, we used a fixed RR model for which the required input events and evaluation labels were available for controlled perturbation. Replacing externally unavailable inputs with reference-partition medians or means affected approximately 1% of RR clinical slots and produced negligible changes in MAE. Source-event removal changed up to 44.79% of slots while retaining RR history, all paired MAE-change intervals included zero (Table 5). This analysis used one fixed RR model and retained RR target history across all conditions.

**Table 5:** Numerical fallback and source-event availability sensitivity in a frozen RR model.

Input condition	MAE [95% interval]	ΔMAE [95% interval]	Perturbed slots/12,528
Observed inputs	3.08135 [2.84943, 3.32316]	Reference	0 (0.00%)
MIMIC-III: item median	3.08135 [2.84943, 3.32316]	7.84 × 10^−7^ [−3.01 × 10^−6^, 4.58 × 10^−6^]	144 (1.15%)
MIMIC-III: item mean	3.08135 [2.84943, 3.32316]	1.13 × 10^−6^ [−2.46 × 10^−6^, 4.73 × 10^−6^]	144 (1.15%)
eICU: item median	3.08135 [2.84943, 3.32316]	8.02 × 10^−7^ [−2.98 × 10^−6^, 4.60 × 10^−6^]	127 (1.01%)
eICU: item mean	3.08135 [2.84943, 3.32316]	1.14 × 10^−6^ [−2.44 × 10^−6^, 4.73 × 10^−6^]	127 (1.01%)
MIMIC-III: remove events	3.08168 [2.85034, 3.32504]	0.00033 [−0.00363, 0.00440]	144 (1.15%)
eICU: remove events	3.07985 [2.84826, 3.32220]	−0.00150 [−0.00453, 0.00130]	127 (1.01%)
Remove most frequent item	3.04091 [2.81353, 3.28374]	−0.04043 [−0.09237, 0.01152]	3,049 (24.34%)
Remove three most frequent items	3.03816 [2.79439, 3.29013]	−0.04319 [−0.14501, 0.06757]	5,611 (44.79%)

ΔMAE is intervention minus baseline (breaths/min), positive values indicate worse accuracy. Intervals use 2,000 shared patient-bootstrap draws. Perturbed slots count masked values for numerical fallback and changed bin inputs after event reselection for source removal. HR, SpO_2_ and temperature were selected by reference-partition frequency. All difference intervals include zero, without establishing equivalence.

### Representative forecast trajectories

We examined individual forecast trajectories to assess the model's behavior in clinical scenarios. Figure 2 presents one example for each of the eight targets, selected as the trajectory whose MAE was closest to the corresponding cohort mean. Overall, the P50 forecasts tracked the main direction of the future trajectories in these examples, while the P10–P90 bands showed target-dependent uncertainty and calibration. In several examples, the bands widened over the forecast horizon. Quantile coverage was closer to the nominal levels for several targets, whereas larger deviations were observed for the upper tail of temperature and the lower tail of the SF ratio (Supplementary Table S3). The P10–P90 bands should therefore be interpreted as target-dependent predictive quantiles rather than uniformly calibrated prediction intervals across all targets and cohorts.

**Figure 2:**
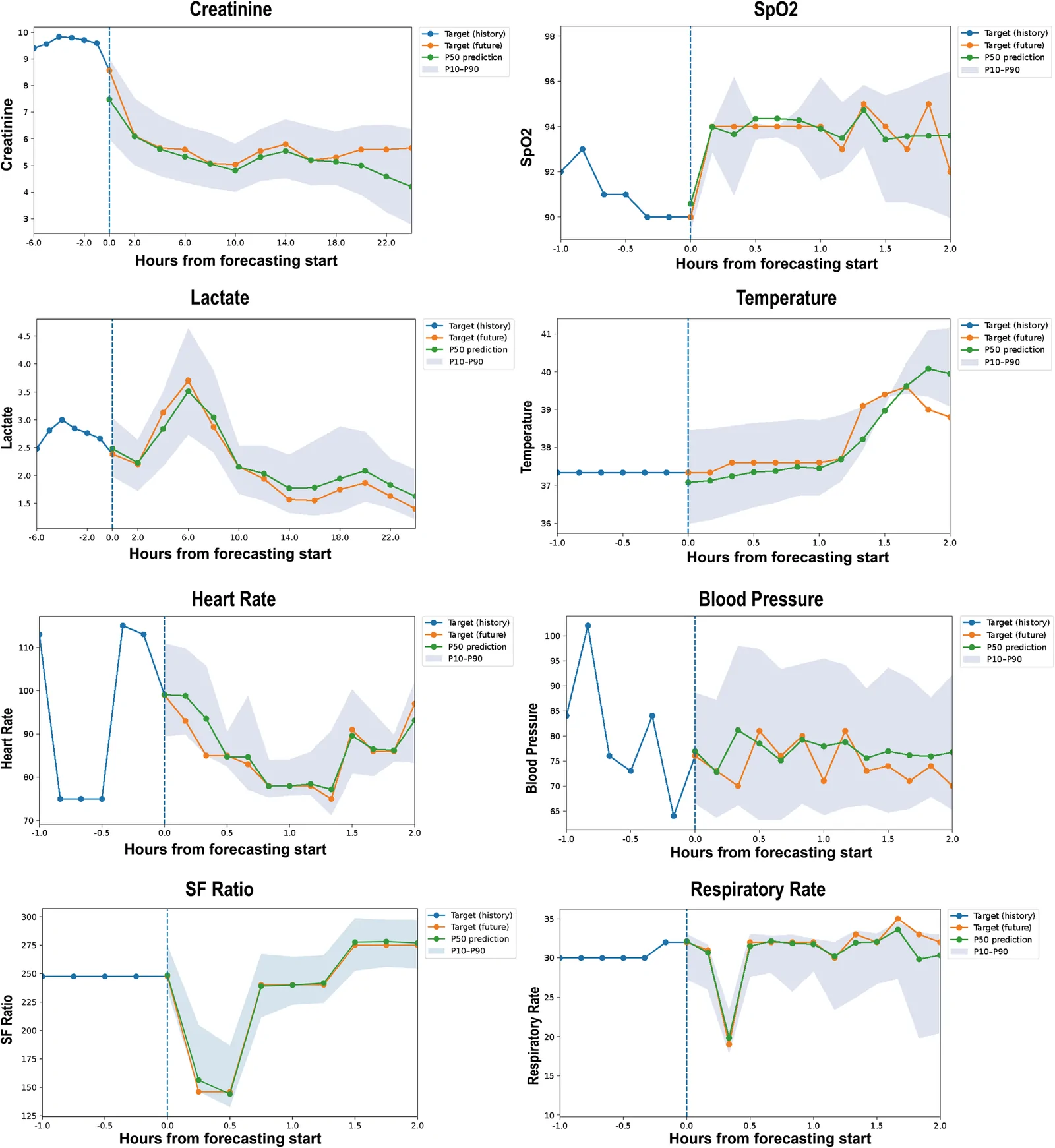
Forecast examples for eight target variables. Representative forecasting trajectories for eight clinical indicators. Each panel shows an illustrative case whose mean absolute error (MAE) for that indicator is closest to the cohort mean, these examples were not selected to demonstrate best-case performance. Blue lines show historical observations (Target history), orange lines show actual future values (Target future), green lines show median predictions (P50), and shaded regions show P10–P90 prediction bands. The vertical dashed line at *t* = 0 marks the prediction start point, with historical data on the left and forecasts on the right. For visualization, only the last 1 h (or 6 h for creatinine and lactate) of the full history is displayed (12 h for vital signs and the SF ratio, 24 h for laboratory targets).

### Model attention in OmniTFT across different time states and feature combinations

To examine the internal decision-making process of OmniTFT, we conducted an analysis of the attention mechanism. Figure 3A shows the attention scores for the 20 features with the highest weights across eight indicators. Figure 3B shows the relative contributions of the five leading features for each target. The largest displayed feature contribution was 44% for blood pressure, 32% for heart rate, and 29% for temperature, compared with 22%–25% for the remaining targets.

**Figure 3:**
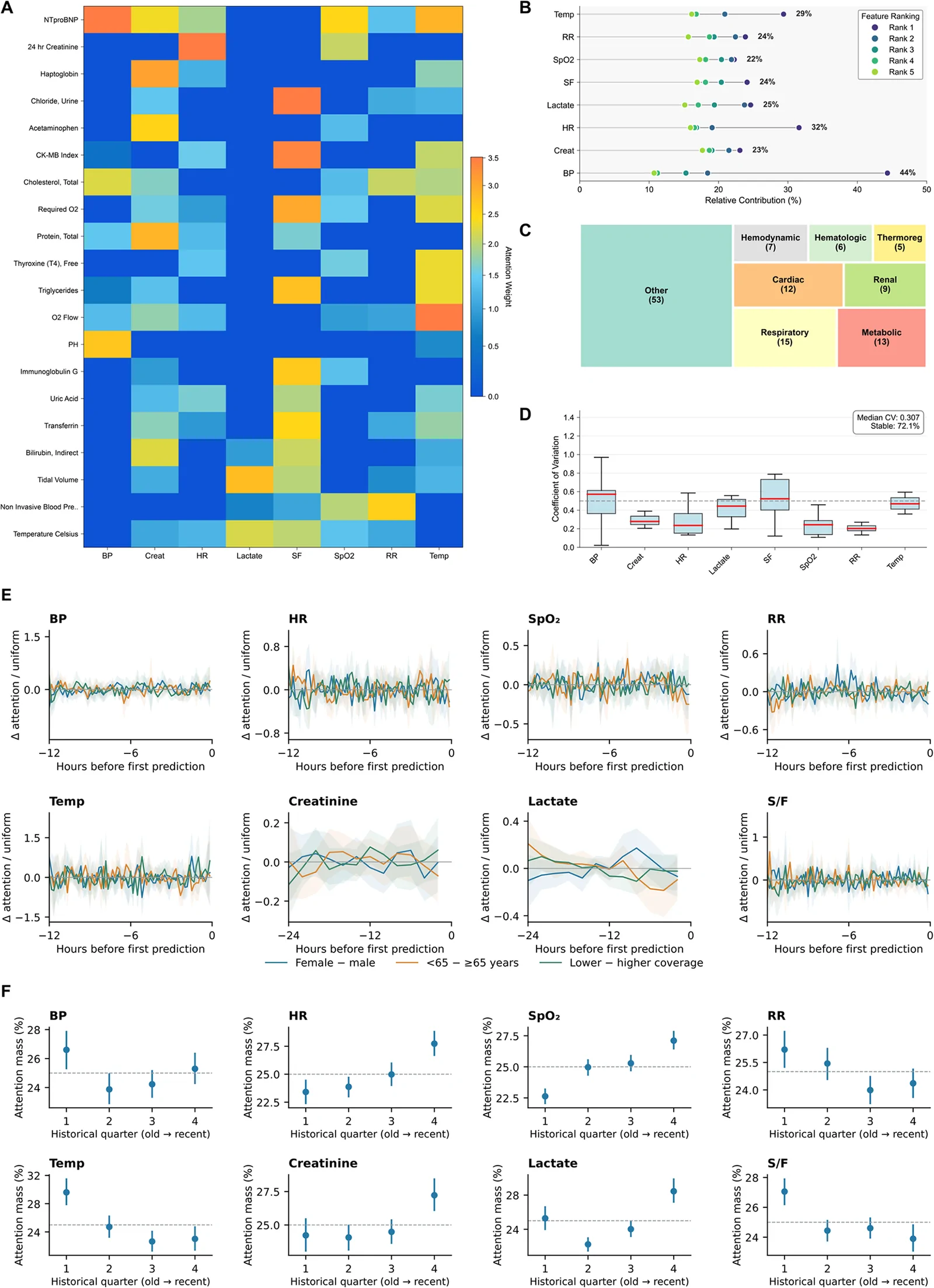
Variable-selection summaries and temporal-attention analyses. **(A)** Heatmap of composite variable-selection scores for the 20 features that appeared most often among the eight per-target Top-15 lists. Color indicates selection strength; cells for targets in whose Top-15 list a feature did not appear are shown in the lowest color and should not be interpreted as zero. BP, blood pressure; Creat, creatinine; HR, heart rate; RR, respiratory rate; Temp, temperature. **(B)** Dot plot of the top five feature contributions per target, dot color denotes rank, and line segments show within-target gradients. **(C)** Distribution of clinical domains among the top 15 feature occurrences for each target, comprising 120 target-specific occurrences in total. **(D)** Distribution of coefficient of variation (CV) across available scores for the top 10 features per target, 68 finite values were available, with a median CV of 0.307 and 72.1% below 0.5. **(E)** Differences in historical temporal-attention profiles between female and male patients, age <65 and ≥65 years, and lower and higher observed-input coverage, expressed relative to uniform attention. **(F)** Historical attention mass across four consecutive quarters of the encoder window, the dashed line denotes 25%. Intervals in E–F were estimated using 2,000 patient-cluster bootstrap resamples. Additional definitions, patient counts, and stability analyses are provided in Supplementary Section S6 and Table S5.

Figure 3C summarizes the clinical domains represented among 120 target-specific Top 15 feature occurrences (15 per target). These selected features span multiple clinical domains rather than being concentrated within a single physiological system. Figure 3D summarizes variability in the available variable-selection scores: the median coefficient of variation was 0.307, and 72.1% of 68 finite values were below 0.5. Patient-bootstrap analyses provided an additional assessment of stability: Spearman agreement with the full-sample variable-selection profiles ranged from 0.909 to 0.985 across targets (Supplementary Table S5).

We additionally examined how temporal attention was distributed across historical positions. Figure 3E compares temporal-attention profiles across sex, age, and observed-input-coverage subgroups, while Figure 3F summarizes attention across four consecutive historical quarters. Subgroup differences in temporal attention fluctuated around uniform allocation (Figure 3E). Because targets use different temporal grids and history lengths, the elapsed duration represented by each quarter differs across targets. Temporal-attention profiles also showed target-dependent allocation across historical positions, with patient-bootstrap Spearman agreement ranging from 0.750 to 0.944 across targets (Supplementary Table S5). Taking together, variable selection and temporal attention describe complementary aspects of model behavior: the former summarizes the relative weighting of recorded clinical inputs, whereas the latter describes how attention is allocated across historical positions (42). These model-derived patterns should not be interpreted as evidence of causal or physiological relationships (36).

### Feature importance analysis using SHAP

To quantify individual feature contributions beyond the general model attention presented in the previous section, we performed SHAP analysis (33). Figure 4A shows mean absolute attributions across target–feature combinations, with CK among the leading features across several targets. Figure 4B shows the signed event-level attribution distribution for HR, illustrating that the contribution assigned to the same feature can vary in magnitude and direction across events. Figure 4C compares mean absolute attribution with the number of targets in which each feature was represented, while Figure 4D summarizes the distribution of attribution values across the available records. Figure 4E further summarizes the number of targets showing positive or negative mean attribution for each feature. Because the model outputs are expressed on target-specific scales, attribution magnitudes should not be directly compared across targets.

**Figure 4:**
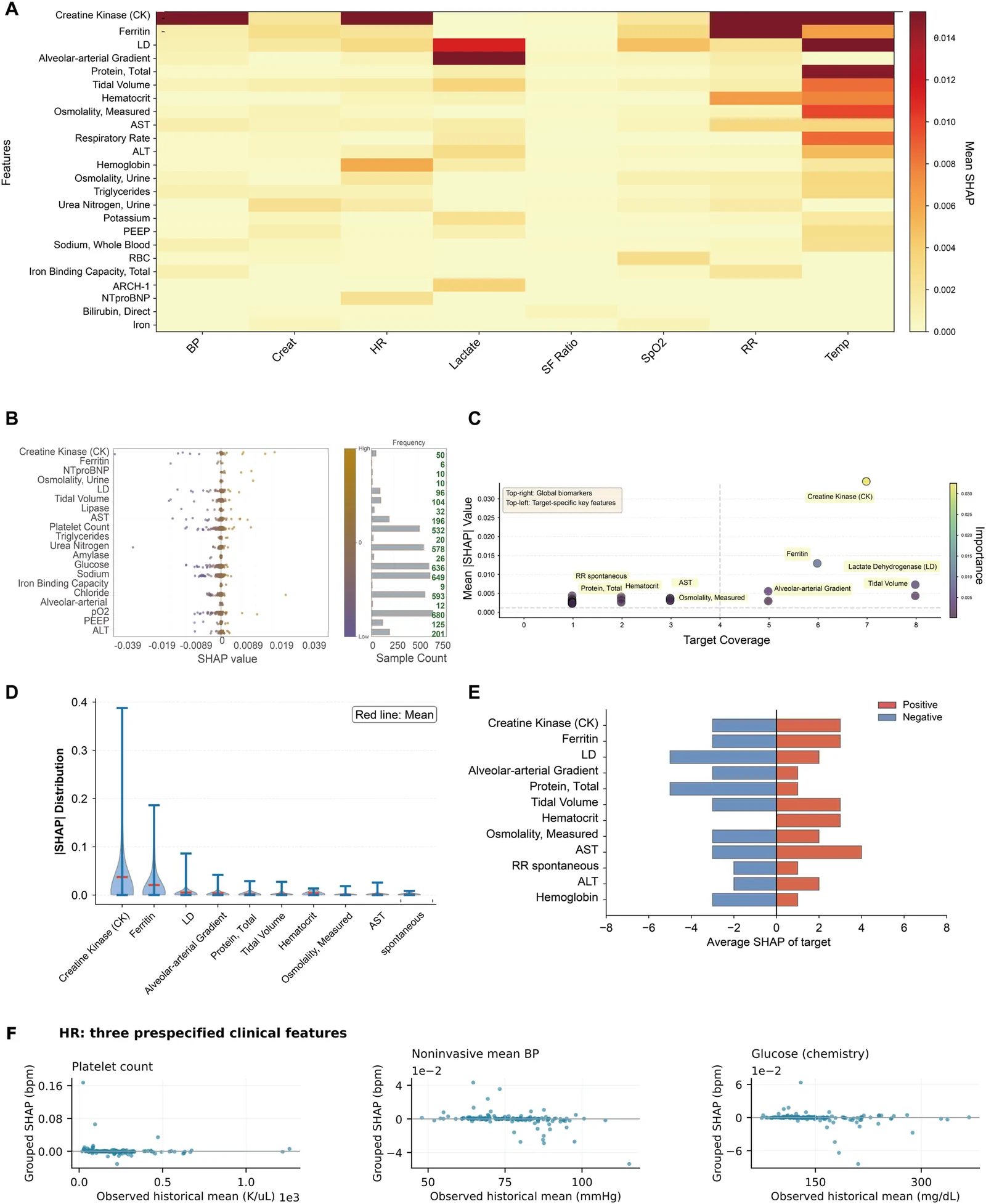
SHAP summaries and HR value associations. **(A)** Heatmap of mean absolute SHAP attribution across features and eight prediction targets, entries unavailable for a target–feature combination should not be interpreted as measured zeros. **(B)** Distribution of signed event-level SHAP attributions for the top 20 features in heart-rate prediction, preserving the displayed feature order. **(C)** Mean absolute attribution in relation to the number of targets for which each feature was represented. **(D)** Distributions of SHAP attribution summaries across the available records. **(E)** Number of targets with positive or negative mean SHAP attribution. **(F)** Grouped-permutation SHAP associations for platelet count, noninvasive mean blood pressure, and chemistry glucose in heart-rate prediction. Each point pairs the mean observed historical value retained in a patient's input window with the attribution of that feature's complete historical value trajectory to the first-step P50 forecast. Supplementary Section S6 and Figures S1–S4 provide additional patient-level distributions, attribution stability, algorithm/background sensitivity, and subgroup analyses.

Figure 4F provides a patient-window analysis for HR, relating retained historical values to grouped SHAP attribution for platelet count, noninvasive mean BP, and chemistry glucose. These stability estimates refer to the grouped patient-window SHAP estimator (Figure 4F and Supplementary Figures S1–S4) rather than to the event-level summaries in Figures 4A–E. Across the eight targets, patient-bootstrap Spearman rank agreement for grouped SHAP ranged from 0.861 to 0.976, with Top 10 Jaccard overlap of 0.667–0.818 (Supplementary Table S5 and Figure S3). The mean retention of the fixed Top 10 features ranged from 80.95% to 92.18% under patient resampling, from 80% to 97% across algorithm seeds, and from 81% to 96% across background draws. Supplementary Figures S1–S4 further report patient-level attribution distributions, historical-value associations, algorithm/background sensitivity, and subgroup comparisons by sex, age, and observed-input coverage. These SHAP results describe reference-dependent model attributions and should not be interpreted as causal or physiological effects.

### Retrospective AKI risk assessment

To examine whether adding an OmniTFT forecast was associated with improved AKI risk prediction, we compared three prediction settings at a single 24 h decision time in the eICU cohort. 374 patients developed incident creatinine-defined stage ≥1 AKI and 45 developed stage ≥2 AKI within the following 24 h. Only serum creatinine was forecast, and the creatinine model generated the full 24 h forecast horizon in a single forward pass without autoregressive iteration. Because the added predictors were generated rather than directly observed future measurements, this downstream analysis was exploratory. For stage ≥2 AKI, AUROC was 0.763 for the forecast-augmented pipeline, compared with 0.693 for Real-24 h and 0.631 for Real-12 h (Figure 5A). In the paired hospital-cluster bootstrap analysis, the AUROC difference vs. Real-24 h was +0.070 (95% interval, 0.022–0.127, DeLong *p* = 0.009, Figure 5G). AUPRC performance showed a similar direction: average precision increased from 0.088 with Real-24 h to 0.127 with the forecast-augmented setting (Figure 5B). AP and Brier score therefore showed favorable point estimates for the forecast-augmented setting, but with greater uncertainty than the AUROC result. With only 45 stage ≥2 events, uncertainty was also wider for calibration and subgroup estimates.

**Figure 5:**
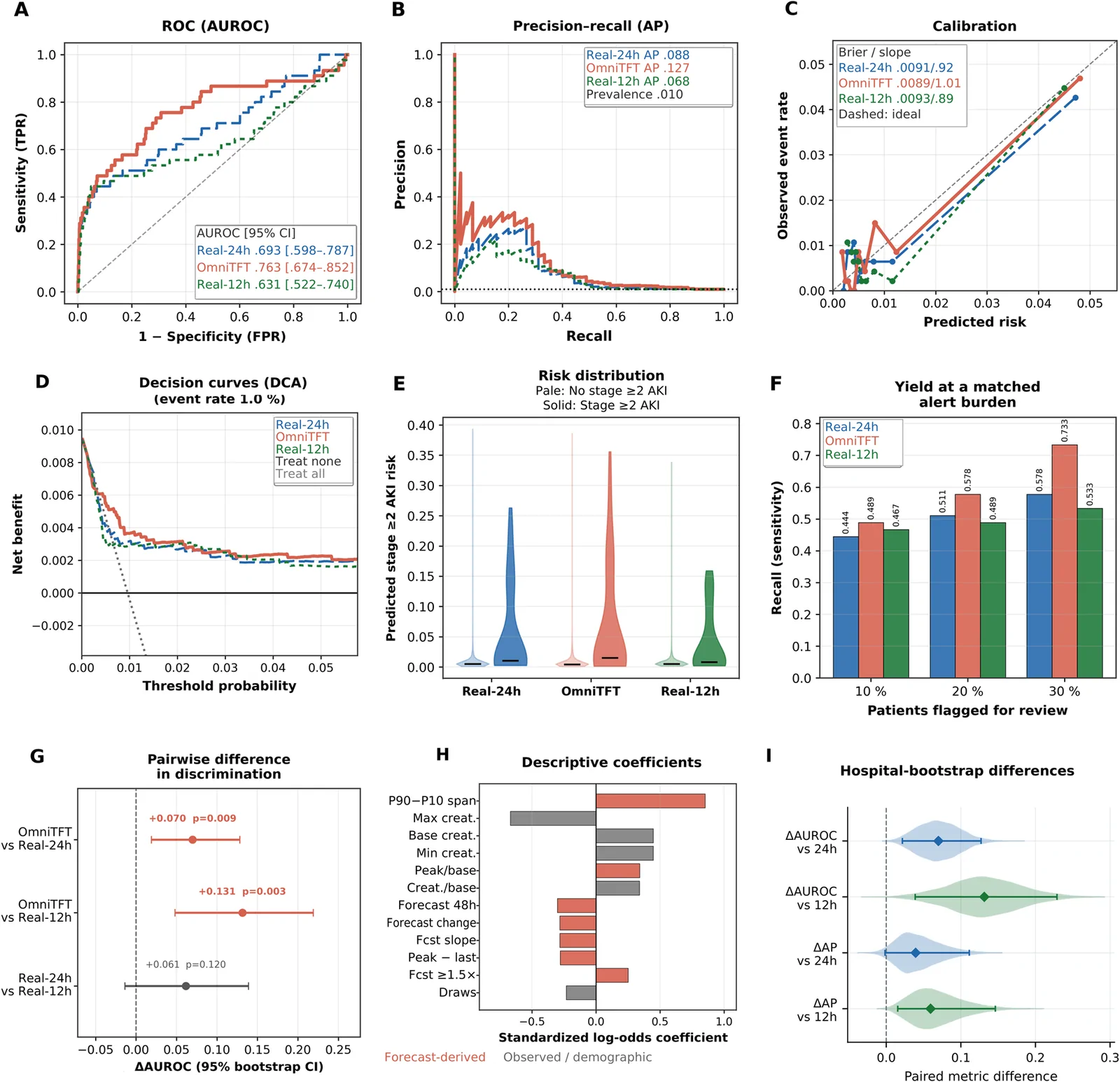
Retrospective prediction of incident creatinine-defined stage ≥2 AKI. The analysis included 4,685 stays with 45 incident stage ≥2 events. Real-24 h, Real-12 h, and the forecast-augmented pipeline are shown using the colors indicated in the figure. **(A)** ROC curves with DeLong 95% confidence intervals. **(B)** Precision–recall curves (AUPRC) and average precision (AP), the dotted line marks the event rate. **(C)** Calibration across ten predicted-risk groups. **(D)** Decision-curve analysis over the evaluated threshold probabilities, with treat-all and treat-none strategies shown for reference. **(E)** Out-of-sample predicted-risk distributions for stage ≥2 events and non-events, each violin is independently normalized and black lines indicate medians. **(F)** Recall when the highest-risk 10%, 20%, or 30% of stays are selected for review. **(G)** Pairwise AUROC differences with stay-level bootstrap intervals and DeLong *P* values. **(H)** Standardized log-odds coefficients from the descriptive forecast-augmented risk model. Forecast change denotes the final minus the first forecast median, and the P90–P10 span denotes max(P90)–min(P10) across the forecast horizon. **(I)** Distributions of AUROC and AP differences from 2,000 paired hospital-cluster bootstrap resamples, diamonds and bars indicate point estimates and percentile 95% intervals. The corresponding numerical estimates and complementary analyses are reported in Table 6 and Supplementary Figure S5.

Predicted risks were generally higher among stays that subsequently developed stage ≥2 AKI than among non-events (Figure 5E). When stays were ranked by predicted risk, reviewing the highest-risk 30% identified 33 of 45 subsequent stage ≥2 events (73.3%) with the forecast-augmented setting, compared with 57.8% for Real-24 h and 53.3% for Real-12 h (Figure 5F). This analysis was also examined at 10% and 20% review thresholds, with the corresponding results shown in Figure 5F and Supplementary Figure S5. The forecast-augmented setting had a calibration slope of 1.01 (95% interval, 0.77–1.25), compared with 0.92 for Real-24 h and 0.89 for Real-12 h. The corresponding Brier scores were 0.0089, 0.0091, and 0.0093, respectively, the Brier-score difference vs. Real-24 h was small and its interval included zero (Figure 5C, Table 6). Decision-curve analysis showed favorable point estimates for the forecast-augmented setting across the evaluated thresholds (Figure 5D). At the 0.5% threshold, the paired net-benefit difference vs. Real-24 h was equivalent to 1.39 additional net true-positive decisions per 1,000 stays (95% interval, 0.29–2.54), whereas the intervals at 1%–3% overlapped zero (Supplementary Figure S5).

**Table 6:** Stage ≥2 AKI risk estimates with paired hospital-bootstrap uncertainty.

Endpoint	Arm or paired difference	AUROC [95% CI]	AP [95% CI]	Brier [95% CI]	Calibration intercept [95% CI]	Calibration slope [95% CI]
Stage ≥2	Real-24 h	0.69264 [0.59755, 0.78723]	0.08762 [0.04204, 0.18788]	0.00910 [0.00682, 0.01148]	0.00469 [−0.30092, 0.26622]	0.91829 [0.65505, 1.16815]
Stage ≥2	OmniTFT	0.76255 [0.67449, 0.85239]	0.12701 [0.05948, 0.25755]	0.00888 [0.00666, 0.01126]	0.01581 [−0.29567, 0.27265]	1.00549 [0.77005, 1.25358]
Stage ≥2	Real-12 h	0.63116 [0.52236, 0.74028]	0.06759 [0.03246, 0.14031]	0.00925 [0.00690, 0.01167]	−0.00578 [−0.30443, 0.23918]	0.89200 [0.56704, 1.18488]
Stage ≥2	OmniTFT minus Real-24 h	0.06990 [0.02162, 0.12712]	0.03938 [−0.00162, 0.11131]	−0.00022 [−0.00047, 0.00001]	0.01111 [−0.03986, 0.06373]	0.08719 [−0.00280, 0.19235]
Stage ≥2	OmniTFT minus Real-12 h	0.13138 [0.03881, 0.22888]	0.05942 [0.01538, 0.14624]	−0.00037 [−0.00070, −0.00008]	0.02159 [−0.02554, 0.06733]	0.11349 [−0.05686, 0.30592]
Stage ≥2	Real-24 h minus Real-12 h	0.06148 [−0.02284, 0.14478]	0.02004 [−0.00521, 0.06619]	−0.00015 [−0.00038, 0.00006]	0.01047 [−0.04379, 0.06676]	0.02629 [−0.13773, 0.18383]

The fixed out-of-fold risks cover 4,685 stays, and 45 events. Calibration intercept fixes slope at one, calibration slope uses a free intercept. AP is non-interpolated average precision. Differences were computed before rounding, higher AUROC/AP and lower Brier are better. AUROC intervals for individual settings are DeLong 95% CIs, all other intervals, including AUROC differences, use 2,000 shared hospital-bootstrap draws without refitting. Figure 5I and Supplementary Figure S5 use these same risks.

In the descriptive coefficient analysis, the forecast P90–P10 span had the largest positive standardized coefficient among the forecast-derived summaries (Figure 5H). As an additional sensitivity analysis with a more common outcome, the secondary stage ≥1 analysis included 374 events and showed a small positive AUROC difference relative to Real-24 h, detailed AP, Brier-score, and uncertainty estimates are reported in Supplementary Figure S5.

Overall, the forecast-augmented prediction setting showed higher discrimination than both observed-window comparators in this retrospective analysis, while the limited number of stage ≥2 events resulted in greater uncertainty for several secondary measures.

## Discussion

Multi-center ICU time series often switch between stable and unstable states. Under these conditions, the attention weights and variable selection scores can be unstable (36, 43), and simple feature selection can lead to omitted variable bias (15, 44). Additionally, long-tailed categorical covariates (45) in EHR data may cause overfitting of rare categories in the embedding, increasing variance, and exacerbating domain shift. OmniTFT addresses these challenges by integrating state-balanced sampling with three regularization mechanisms designed for these EHR characteristics. The ablation analysis showed increased prediction error after removal of each proposed component, although the magnitude of the effect varied across targets. Removing state-balanced sampling produced the largest MAE increases for BP, HR, SpO_2_, and RR, whereas removing group regularization produced the largest increases for the remaining targets. Within the tested settings, the reference threshold and sampling configuration had the lowest mean MAE across all targets, these fluctuation thresholds are modeling choices for sampling rather than clinical diagnostic criteria.

Our results demonstrate that OmniTFT can model multiple vital-sign and laboratory forecasting tasks across three ICU databases within the study's disease-enriched cohorts, with competitive performance across targets. This cross-cohort performance suggests that OmniTFT may serve as an auxiliary forecasting module in ICU clinical information systems. As a multi-target forecasting model, OmniTFT provides probabilistic forecasts and uncertainty estimates for multiple physiological variables through shared model parameters and a consistent preprocessing and inference pipeline. Because different targets retain clinically appropriate temporal grids, their forecasts are generated through target-specific forward passes within the same shared-backbone framework rather than through a single simultaneous forward pass. From a systems perspective, this capability facilitates integration into dashboards and early warning workflows based on EHR. Practical deployment also requires target-specific checkpoints, scalers, and variable dictionaries, together with causal timestamp alignment, unit and range checks, and local calibration and alert-threshold evaluation. The computational measurements reported here characterize the evaluated implementation, while complete clinical-system latency will additionally depend on data extraction, preprocessing, model loading, and system integration.

A key consideration for deployment in clinical systems is whether model behavior is amenable to clinical interpretation. Our variable-selection, temporal-attention, and SHAP analyses identified target-dependent patterns spanning multiple physiological systems. In addition to target-associated variables, several features, including creatine kinase, ferritin, and lactate dehydrogenase, were represented across multiple prediction targets, while attribution patterns varied across targets and records. These findings suggest that OmniTFT adjusts its reliance on individual features according to the prediction target and patient context. However, these importance patterns represent statistical associations learned from the EHR data and should not be interpreted as evidence of causal physiological relationships. Additional patient-resampling, algorithm sensitivity, and subgroup analyses across sex, age, and observed-input coverage were used to characterize the stability of these fitted-model associations.

Despite the advantages described above, several challenges remain. First, predictive performance remained target-dependent. Compared with the other models, OmniTFT ranked second for creatinine and respiratory rate and third for temperature, indicating that the shared-backbone approach did not outperform all comparator models for every target. We suspect that sampling leaves limited joint samples between features and the target within the window, weakening the available signal. A larger and more densely observed cohort may help mitigate this limitation (46). Second, OmniTFT development benefited from a broad and heterogeneous input feature set, allowing the framework to learn clinically useful within-window feature combinations across diverse physiological contexts. At deployment, however, inference does not require manual construction of target-specific feature subsets. Instead, OmniTFT can operate on routinely collected variables available within the current observation window, without requiring manual construction of target-specific feature subsets. Variables unavailable in an external cohort were imputed with MIMIC-IV training medians, a population-level fallback that has performed comparably to more complex imputation approaches in clinical prediction models (30). In the targeted RR sensitivity analysis, which isolated the effect of external input availability under otherwise unchanged evaluation conditions, replacing externally unavailable inputs with medians or means and removing the corresponding source events produced no clear change in MAE (Table 5), suggesting that this imputation strategy is unlikely to explain the differences in external performance for this target. Because the analysis examined a single target with its history retained, the influence on other targets was not directly quantified. The analysis varied the fallback value and the availability of clinical inputs, but not causal forward filling, the window-exclusion rule or missingness in the target history, and median imputation cannot recover unmeasured patient-specific information (28). Third, probabilistic calibration was target- and cohort-dependent. Although empirical P10 and P90 coverage was close to the nominal levels for several targets, larger deviations were observed after transfer to external cohorts, including reduced upper-tail coverage for temperature and miscalibration of the lower SF-ratio quantile. Accordingly, the P10–P90 outputs should be interpreted as target-based uncertainty estimates rather than universally calibrated clinical prediction intervals. Target and site-specific recalibration should therefore be evaluated before prospective clinical deployment. Finally, because the study cohorts were enriched for patients with sepsis, ARDS, or AKI, the generalizability of OmniTFT to an unselected ICU population remains to be established.

The retrospective AKI analysis has additional limitations. Only 45 of 4,685 eligible stays developed the primary stage ≥2 outcome, resulting in wider uncertainty for AP, calibration, and subgroup estimates (47). AP, Brier-score, calibration, hospital-bootstrap, decision-curve, and secondary stage ≥1 analyses were therefore included to provide complementary assessments of the findings. Several secondary estimates remained uncertain, including AP and Brier-score differences vs. Real-24 h and decision-curve differences at thresholds of 1%–3%. Because the downstream models were selected independently for each prediction setting, this analysis compares forecast-augmented and observed-record-only pipelines rather than the effect of adding forecasts to a single fixed risk model.

Future work could extend OmniTFT to incorporate continuous physiological waveforms (e.g., ECG, arterial line tracings), which capture high-frequency dynamics not captured by the discretely resampled EHR variables used here (48, 49). In addition, prospective validation in a live clinical workflow would be necessary to assess real-world utility, alert fatigue, and clinician acceptance. Together, these advances have the potential to further enhance predictive models, however, careful validation using data from multiple modalities is essential to ensure robustness across diverse clinical settings and patient populations.

Overall, the results support further evaluation of OmniTFT as a common multi-target forecasting framework for ICU EHR data, while broader external and prospective validation remains necessary before clinical deployment.

## Conclusions

In this study, we developed OmniTFT, a unified ICU forecasting model for vital signs, laboratory results, and derived indicators within a shared architecture and training workflow. This model facilitates consistent development, evaluation, and extension across heterogeneous ICU forecasting tasks while retaining target-specific temporal grids and output heads within a common inference framework. By generating multi-target forecasts directly from routinely collected EHR data, OmniTFT has the potential to serve as a forecasting layer for ICU information systems and dashboard-based decision support, pending further prospective, and implementation-focused validation.

## Data Availability

The datasets used in this study are publicly available through PhysioNet (https://physionet.org/) upon completion of required training and approval: MIMIC-IV v2.2 (Medical Information Mart for Intensive Care IV): Available at: https://physionet.org/content/mimiciv/2.2/. This database contains deidentified health data from Beth Israel Deaconess Medical Center (2008-2019). MIMIC-III CareVue v1.4 (Medical Information Mart for Intensive Care III): Available at: https://physionet.org/content/mimic3-carevue/1.4/ This database contains deidentified health data from Beth Israel Deaconess Medical Center (2001-2008). eICU CRD v2.0 (eICU Collaborative Research Database): Available at: https://physionet.org/content/eicu-crd/2.0/. This multicenter database contains deidentified health data from 208 hospitals across the United States (2014-2015). Access to these databases requires completion of the CITI “Data or Specimens Only Research” training course and signing of a data use agreement. All datasets are governed by the Health Insurance Portability and Accountability Act (HIPAA) and comply with deidentification standards. No additional institutional review board approval was required for this study as all data is publicly available and de-identified. The relevant code for OmniTFT implementation can be found in the Git repository at the following link: https://github.com/WanzheUTokyo/OmniTFT-Fais.
