# Supplementary material for: OmniTFT: an EHR-driven multi-target forecasting model for ICU clinical decision support systems
Source: Front Digit Health. 2026 Oct 16;8:1965956. doi: 10.3389/fdgth.2026.1965956 (PMC13719366; doi:10.3389/fdgth.2026.1965956)
Supplement: Supplementary file 1 [file Datasheet1.docx]

**Supplementary material for:**

**OmniTFT: An EHR-Driven Multi-Target Forecasting Model for ICU Clinical Decision Support Systems**

# **Contents**

S1. Cohorts and clinical variables

S2. Model formulation and training

S3. Forecast performance and comparator evaluation

S4. Component ablation and sampling sensitivity

S5. Input availability and missingness sensitivity

S6. Attention and SHAP analyses

S7. Forecast-augmented AKI analysis

S8. Computational cost and deployment

Supplementary references

Supplementary Figures S1–S5

# **S1. Cohorts and clinical variables**

**Table S1. Baseline characteristics of the MIMIC-IV, MIMIC-III CareVue and eICU cohorts**

| Characteristic | MIMIC-IV | MIMIC-III | eICU |
| --- | --- | --- | --- |
| Total Admissions | 13277 | 4188 | 23238 |
| Gender, n (%) |  |  |  |
| Male | 7720 (58.1%) | 2340 (55.9%) | 12274 (52.8%) |
| Female | 5557 (41.9%) | 1848 (44.1%) | 10964 (47.2%) |
| Age (years), mean (SD) | 64.45 (15.14) | 65.25 (16.46) | 62.84 (12.77) |
| Height (cm), mean (SD) | 169.30 (11.38) | 163.93 (15.34) | 168.95 (10.98) |
| Weight (kg), mean (SD) | 87.07 (24.20) | 80.29 (26.52) | 83.99 (27.10) |
| Race, n (%) |  |  |  |
| White | 8997 (67.8%) | 2933 (70.0%) | 17874 (76.9%) |
| Black/African American | 2084 (15.7%) | 391 (9.3%) | 2511 (10.8%) |
| Asian | 328 (2.5%) | 95 (2.3%) | 408 (1.8%) |
| Hispanic/Latino | 592 (4.5%) | 90 (2.1%) | 1031 (4.4%) |
| American Indian/Alaska Native | 48 (0.4%) | 3 (0.1%) | 183 (0.8%) |
| Native Hawaiian/Pacific Islander | 15 (0.1%) | 1 (0.0%) | - |
| Portuguese | 73 (0.5%) | 1 (0.0%) | - |
| Middle Eastern | - | 2 (0.0%) | - |
| Multiple Race/Ethnicity | 9 (0.1%) | 3 (0.1%) | - |
| Unknown/Declined | 807 (6.1%) | 594 (14.2%) | 158 (0.7%) |
| Other | 324 (2.4%) | 75 (1.8%) | 1073 (4.6%) |

*SD: standard deviation, "-": data not available or category is not represented in the database. Race categories include merged subcategories: White (includes all White subgroups), Black/African American (includes all Black/African subgroups), Asian (includes all Asian subgroups), Hispanic/Latino (includes all Hispanic/Latino subgroups and South American), Unknown/Declined (includes Unknown, Unable to Obtain, and Patient Declined to Answer).*

**Table S2. Clinical Variables**

| **Variable (Unit)** | **MIMIC-IV** | **MIMIC-III** | **eICU** |
| --- | --- | --- | --- |
| Amylase (IU/L) | 127.81 (187.38) | 115.65 (196.16) | 142.15 (237.54) |
| Heart Rate (bpm) | 89.03 (18.79) | 88.72 (18.47) | 88.97 (18.92) |
| Mean Airway Pressure (cmH2O) | 10.02 (4.11) | - | - |
| Total Protein, Urine (mg/dL) | 126.02 (242.51) | 147.76 (331.45) | 131.75 (250.08) |
| Lactate Dehydrogenase (LD) (IU/L) | 641.34 (442.83) | 618.85 (549.18) | 659.56 (424.42) |
| GCS - Verbal Response (score) | 3.22 (1.59) | - | - |
| Creatinine (mg/dL) | 1.89 (1.52) | 1.84 (1.50) | 1.99 (1.60) |
| White Blood Cells (K/uL) | 12.67 (8.50) | 12.56 (8.71) | 13.06 (9.05) |
| GCS - Motor Response (score) | 5.50 (1.42) | - | - |
| Sodium, Urine (mEq/L) | 53.02 (36.69) | 53.94 (36.34) | 55.58 (38.00) |
| Venous CO2 Pressure (mmHg) | 45.15 (10.14) | - | - |
| Arterial Blood Pressure mean (mmHg) | 76.95 (14.95) | 77.20 (15.20) | 77.21 (14.85) |
| Potassium (mEq/L) | 4.17 (0.65) | 4.06 (0.61) | 4.11 (0.56) |
| Minute Volume (L/min) | 9.70 (3.09) | - | - |
| Ferritin (ng/mL) | 2763.87 (13136.76) | 3554.40 (14336.43) | 4670.48 (19724.85) |
| Free Calcium (mmol/L) | 1.11 (0.11) | 1.11 (0.11) | 1.12 (0.12) |
| Sodium, Whole Blood (mEq/L) | 135.63 (6.14) | 135.46 (6.91) | 133.82 (7.48) |
| Sedimentation Rate (mm/hr) | 67.79 (41.22) | 71.37 (43.53) | 55.50 (40.60) |
| Cholesterol Ratio (Total/HDL) (Ratio) | 4.89 (4.21) | 4.95 (4.09) | 4.49 (3.10) |
| Red Blood Cells (m/uL) | 3.10 (0.62) | 3.08 (0.63) | 3.09 (0.62) |
| Temperature (°C) | 37.06 (0.73) | 37.09 (1.07) | 36.99 (0.96) |
| Hemoglobin, CBC (g/dL) | 9.50 (2.11) | - | - |
| Oxygen (%) | 53.41 (19.65) | 53.52 (19.34) | 54.16 (19.83) |
| CK-MB Index (%) | 5.89 (6.06) | 5.79 (6.02) | 6.54 (5.70) |
| Oxygen Saturation, Blood Gas (%) | 97.20 (2.80) | 97.10 (3.50) | 97.15 (2.90) |
| Urine pH (units) | 5.88 (0.74) | 5.89 (0.75) | 5.87 (0.73) |
| Arterial O2 pressure (mmHg) | 95.30 (25.25) | - | - |
| Osmolality, Urine (mOsm/kg) | 398.31 (134.19) | 398.27 (135.42) | 381.22 (119.76) |
| Alveolar-arterial Gradient (mm Hg) | 176.42 (112.35) | 188.37 (128.64) | 215.68 (145.09) |
| Potassium, Urine (mEq/L) | 40.10 (20.66) | 39.12 (20.09) | 38.37 (19.79) |
| Triglycerides (mg/dL) | 268.05 (354.01) | 251.77 (272.97) | 254.27 (456.51) |
| Troponin T (ng/mL) | 1.96 (4.68) | 1.75 (4.04) | 1.26 (3.08) |
| O2 Flow, Standard (L/min) | 6.01 (3.50) | - | - |
| pCO2 (mm Hg) | 42.10 (10.08) | 42.46 (11.49) | 41.66 (11.07) |
| Creatine Kinase (CK) (IU/L) | 750.88 (1601.59) | 629.35 (1493.23) | 735.01 (1589.85) |
| Bilirubin, Indirect (mg/dL) | 2.17 (2.67) | 2.48 (3.22) | 2.49 (2.90) |
| Anion Gap (mEq/L) | 12.15 (4.39) | 10.08 (4.50) | 9.51 (4.35) |
| GCS - Eye Opening (score) | 3.49 (1.10) | - | - |
| Albumin (g/dL) | 2.89 (0.64) | 2.85 (0.65) | 2.91 (0.65) |
| RDW (%) | 16.94 (2.85) | 16.99 (2.89) | 17.01 (2.84) |
| Inspiratory Time (sec) | 0.85 (0.31) | - | - |
| RDW-SD (fL) | 56.45 (10.61) | 56.81 (10.68) | 55.93 (10.09) |
| PT (sec) | 18.68 (9.73) | 18.87 (14.12) | 18.93 (9.75) |
| pO2 (mm Hg) | 117.69 (32.69) | 116.98 (32.44) | 117.15 (31.08) |
| Creatinine, Urine (mg/dL) | 85.32 (58.99) | 80.55 (57.41) | 83.74 (60.39) |
| Acetaminophen (ug/mL) | 23.18 (32.49) | 10.77 (6.41) | 25.73 (31.44) |
| Globulin (g/dL) | 2.51 (0.98) | 2.52 (0.82) | 2.49 (0.87) |
| Sodium (mEq/L) | 138.77 (5.88) | 136.94 (5.85) | 135.62 (8.11) |
| eAG (mg/dL) | 150.25 (59.24) | 158.87 (70.32) | 143.22 (52.48) |
| Peak Insp. Pressure (cmH2O) | 22.23 (8.39) | - | - |
| Bilirubin, Direct (mg/dL) | 0.75 (1.70) | 0.80 (1.80) | 0.85 (1.90) |
| Hematocrit (%) | 28.24 (5.07) | 28.19 (5.08) | 28.18 (5.10) |
| Spont RR (bpm) | 22.09 (7.29) | - | - |
| Thyroxine (T4), Free (ng/dL) | 1.17 (0.74) | 1.23 (0.76) | 1.10 (0.37) |
| O2 Flow, Ventilator (L/min) | 13.44 (6.50) | 13.33 (5.85) | 15.80 (7.09) |
| C-Reactive Protein (mg/L) | 104.10 (77.82) | 84.86 (80.42) | 96.81 (77.04) |
| Thyroid Stimulating Hormone (uIU/mL) | 4.77 (9.52) | 3.75 (6.43) | 3.57 (4.64) |
| Alkaline Phosphatase (IU/L) | 162.09 (164.61) | 174.42 (197.28) | 156.02 (173.07) |
| Vitamin B12 (pg/mL) | 937.80 (470.90) | 949.19 (479.55) | 938.13 (461.87) |
| Aspartate Aminotransferase (AST) (IU/L) | 273.91 (1130.96) | 281.65 (1149.27) | 306.81 (1280.51) |
| Lipase (IU/L) | 149.04 (346.36) | 145.00 (385.99) | 159.19 (332.43) |
| Cuff Pressure (cmH2O) | 27.01 (4.78) | - | - |
| Respiratory Rate (Total) (insp/min) | 22.21 (6.48) | 22.40 (6.57) | 21.64 (5.99) |
| Iron (ug/dL) | 49.67 (45.25) | 50.63 (46.57) | 52.14 (47.07) |
| Central Venous Pressure (mmHg) | 16.37 (3.52) | - | - |
| Glucose, Serum/Plasma (mg/dL) | 138.34 (53.03) | 138.91 (53.82) | 140.00 (70.00) |
| Transferrin (mg/dL) | 154.43 (63.09) | 154.39 (63.17) | 151.80 (61.69) |
| Glucose, Blood Gas (mg/dL) | 145.50 (62.00) | 146.00 (65.00) | 146.50 (68.00) |
| Cholesterol, Total (mg/dL) | 129.31 (58.98) | 135.98 (67.78) | 128.80 (52.43) |
| Tidal Volume (observed) (mL) | 465.04 (103.60) | - | - |
| Haptoglobin (mg/dL) | 182.59 (122.00) | 179.67 (126.82) | 166.69 (122.70) |
| Chloride (mEq/L) | 102.39 (7.30) | 102.70 (7.16) | 102.21 (7.45) |
| Potassium, Whole Blood (mEq/L) | 4.17 (0.77) | 4.17 (0.73) | 4.22 (0.83) |
| Urea Nitrogen (mg/dL) | 39.83 (26.69) | 39.77 (26.84) | 39.94 (27.40) |
| Total PEEP Level (cmH2O) | 9.04 (3.30) | - | - |
| Calcium, Total (mg/dL) | 8.46 (0.89) | 8.25 (0.38) | 8.54 (1.92) |
| Hemoglobin, Blood Gas (g/dL) | 9.20 (1.73) | 9.09 (2.13) | 8.99 (2.72) |
| Hemoglobin, Point of Care (g/dL) | 9.63 (2.45) | 9.60 (1.97) | 9.53 (1.99) |
| Creatine Kinase, MB Isoenzyme (ng/mL) | 18.91 (47.35) | 18.62 (44.99) | 20.07 (50.74) |
| Respiratory Rate (insp/min) | 20.92 (5.87) | 21.07 (5.87) | 20.69 (5.82) |
| Tidal Volume (spontaneous) (mL) | 480.30 (152.24) | - | - |
| Lactate (mmol/L) | 2.62 (2.47) | 2.56 (2.33) | 2.61 (2.45) |
| PEEP (cmH2O) | 8.84 (3.21) | 8.76 (3.03) | 9.24 (3.20) |
| Nucleated Red Cells (%) | 4.50 (12.85) | 4.22 (11.30) | 6.11 (17.41) |
| Chloride, Whole Blood (mEq/L) | 104.32 (7.29) | 103.86 (7.12) | 103.96 (7.38) |
| Required O2 (%) | 52.59 (18.22) | 52.50 (18.37) | 53.64 (17.81) |
| Cholesterol, LDL, Calculated (mg/dL) | 69.05 (42.32) | 67.51 (33.06) | 64.56 (34.48) |
| Vancomycin (ug/mL) | 19.08 (8.31) | 19.11 (7.81) | 19.76 (8.74) |
| Arterial Blood Pressure systolic (mmHg) | 116.82 (23.01) | 117.19 (23.08) | 115.78 (22.69) |
| Hematocrit, Calculated (%) | 28.92 (7.34) | 28.86 (5.92) | 28.33 (5.43) |
| Non Invasive Blood Pressure mean (mmHg) | 83.53 (14.95) | 83.09 (15.20) | 83.77 (14.85) |
| Venous O2 Pressure (mmHg) | 35.26 (8.98) | - | - |
| Specific Gravity (-) | 1.01 (0.01) | - | 1.02 (0.01) |
| Iron Binding Capacity, Total (ug/dL) | 200.80 (82.01) | 200.75 (82.12) | 197.38 (80.20) |
| Inspired Gas Temp. (°C) | 36.55 (2.20) | - | - |
| Immunoglobulin A (mg/dL) | 299.30 (140.36) | 297.17 (145.47) | 303.32 (131.97) |
| INR(PT) (-) | 1.72 (0.95) | 1.73 (0.93) | 1.74 (0.92) |
| Arterial O2 Saturation (%) | 95.45 (5.15) | - | - |
| Folate (ng/mL) | 11.56 (4.18) | 11.79 (4.39) | 11.70 (4.37) |
| Non Invasive Blood Pressure diastolic (mmHg) | 65.21 (12.86) | 64.78 (13.24) | 65.34 (12.91) |
| Norepinephrine (mcg/kg/min) | 0.12 (0.18) | - | - |
| Phosphate (mg/dL) | 3.83 (1.55) | 3.82 (1.56) | 3.87 (1.59) |
| Respiratory Rate (spontaneous) (insp/min) | 11.35 (8.08) | 10.93 (9.11) | 10.61 (8.56) |
| Protein/Creatinine Ratio (mg/mg) | 2.25 (5.23) | 3.00 (16.11) | 2.34 (4.95) |
| RBC (#/hpf) | 26.12 (67.27) | 28.49 (76.79) | 26.32 (68.07) |
| Plateau Pressure (cmH2O) | 21.53 (7.25) | - | - |
| Cortisol (ug/dL) | 25.82 (25.71) | 26.12 (25.69) | 25.63 (23.88) |
| NTproBNP (pg/mL) | 9740.26 (12092.67) | 10658.70 (12935.68) | 9471.15 (11485.19) |
| Platelet Count (K/uL) | 198.61 (140.90) | 196.74 (146.49) | 191.60 (141.98) |
| Glucose, Whole Blood (mg/dL) | 143.94 (70.67) | 143.66 (75.97) | 143.91 (79.86) |
| MCV (fL) | 92.06 (7.31) | 92.30 (7.05) | 92.24 (7.32) |
| Magnesium (mg/dL) | 2.14 (0.44) | 2.13 (0.51) | 2.13 (0.38) |
| Chloride, Urine (mEq/L) | 52.73 (39.56) | 53.81 (40.20) | 54.46 (40.41) |
| Bilirubin, Total (mg/dL) | 1.85 (3.40) | 1.95 (3.60) | 2.05 (3.80) |
| O2 saturation pulseoxymetry (%) | 96.94 (3.01) | 95.86 (2.97) | 96.41 (2.02) |
| Osmolality, Measured (mOsm/kg) | 305.62 (22.68) | 307.56 (21.64) | 307.39 (20.24) |
| Protein, Total (g/dL) | 5.53 (1.05) | 5.51 (1.05) | 5.45 (0.96) |
| Inspired O2 Fraction (%) | 48.57 (15.39) | 49.04 (17.20) | 49.29 (16.95) |
| Cholesterol, HDL (mg/dL) | 34.70 (17.39) | 35.61 (18.50) | 35.83 (17.04) |
| Bicarbonate (mEq/L) | 24.23 (5.57) | 24.16 (5.54) | 23.90 (5.67) |
| Arterial Blood Pressure diastolic (mmHg) | 57.26 (12.91) | 57.51 (13.14) | 57.91 (12.78) |
| Base Excess (mEq/L) | -0.73 (6.49) | -0.77 (6.09) | -1.05 (7.44) |
| Immunoglobulin M (mg/dL) | 115.93 (114.29) | 103.89 (112.95) | 95.42 (109.83) |
| % Hemoglobin A1c (%) | 6.89 (2.06) | 7.12 (2.38) | 6.72 (1.97) |
| Calculated Total CO2 (mEq/L) | 25.51 (6.58) | 25.45 (6.52) | 25.07 (6.45) |
| Alanine Aminotransferase (ALT) (IU/L) | 182.03 (639.90) | 199.38 (674.96) | 220.07 (694.24) |
| WBC (#/hpf) | 22.32 (51.19) | 22.95 (54.72) | 23.43 (55.71) |
| MCHC (%) | 32.51 (1.75) | 32.42 (1.60) | 31.71 (1.11) |
| Non Invasive Blood Pressure systolic (mmHg) | 120.18 (21.84) | 119.72 (22.31) | 120.64 (21.57) |
| 24 hr Creatinine (mg/24hr) | 715.47 (492.96) | 793.27 (521.26) | 674.21 (587.54) |
| MCH (pg) | 29.89 (2.60) | 29.98 (2.55) | 29.96 (2.70) |
| Urea Nitrogen, Urine (mg/dL) | 465.82 (277.00) | 465.01 (279.91) | 444.71 (263.49) |
| Blood Temperature CCO (C) (°C) | 37.05 (0.68) | 37.09 (0.73) | 37.03 (0.77) |
| Tidal Volume (mL) | 448.60 (113.59) | 447.08 (137.62) | 447.34 (120.78) |
| Parathyroid Hormone (pg/mL) | 159.76 (233.82) | 154.42 (206.66) | 203.83 (173.64) |
| Uric Acid (mg/dL) | 5.55 (3.35) | 5.39 (2.96) | 5.77 (3.78) |
| pH (units) | 7.37 (0.09) | 7.36 (0.10) | 7.38 (0.08) |
| PTT (sec) | 48.76 (26.01) | 49.08 (25.97) | 50.38 (27.57) |
| Immunoglobulin G (mg/dL) | 1011.37 (422.35) | 932.19 (400.27) | 1067.75 (415.51) |
| Urobilinogen (mg/dL) | 2.31 (1.92) | 2.38 (1.84) | 2.33 (1.99) |

*The table lists 140 clinical-variable entries, source- and unit-specific channels are retained separately. Demographic and admission covariates (age, height, weight, gender, race and admission type) are summarized in Table S1. "-" indicates that the corresponding item was not retrieved by our harmonization pipeline for that database, not that the measurement is absent from the source database, in external validation, these channels were treated as missing and imputed with the MIMIC-IV training-set median.*

# **S2. Model formulation and training**

## **S2.1. State-balanced sliding window sampler**

First, align each patient's slices to a fixed time grid to generate a chronologically ordered target sequence $\mathbf{y}_{\mathbf{1}:\mathbf{n}}$. We then slide windows of length $\mathbf{T} = \mathbf{E} + \mathbf{H}$ with encoder length E and horizon length H over the sequence. For a window starting at index $\mathbf{t}\in\{\mathbf{1},\ldots,\mathbf{n}-\mathbf{T}+\mathbf{1}\}$ the encoder segment is $\boldsymbol{y}_{\boldsymbol{t}:\boldsymbol{t}+\boldsymbol{E}-\boldsymbol{1}}$. and the future segment is $\boldsymbol{y}_{\boldsymbol{t}+\boldsymbol{E}:\boldsymbol{t}+\boldsymbol{T}-\boldsymbol{1}}$. We determine the relationship between fluctuations and physiological change thresholds by calculating the future segment fluctuation score $\boldsymbol{S}_{\boldsymbol{t}}$ as:

$$\begin{aligned} S_{t}=\max_{\tau\in\left[ t+E,t+T-1 \right]}y_{\tau}-\min_{\tau\in\left[ t+E,t+T-1 \right]}y_{\tau}\quad\left( S-1 \right) \end{aligned}$$

The score retains the same unit as the target and is simply the max minus min over the future window. We then set a prespecified modeling cutoff $\boldsymbol{\delta}$ for that target. For the state-balanced sampler, the target-specific volatility threshold $\boldsymbol{\delta}$ was fixed a priori and held constant across MIMIC-IV: blood pressure 10 mmHg, heart rate 15 bpm, SpO2 3 percentage points, respiratory rate 5 breaths/min, temperature 0.5°C, creatinine 0.3 mg/dL, lactate 1.0 mmol/L, and SF ratio 30. A window was labeled volatile when max(yfuture) − min(yfuture) > $\boldsymbol{\delta}$ and stable otherwise. These cutoffs were chosen to represent short-horizon changes that are clinically meaningful rather than monitor-level noise; they define training states and are not diagnostic criteria. The creatinine cutoff shares a numerical value with one KDIGO criterion but is applied to a different quantity and window. The sensitivity analysis varied each cutoff to 0.5× and 1.5× its reference value while retaining 50% volatile-window sampling (Table 4); validation, test and external evaluation retained the naturally occurring distribution of stable and volatile windows.

## **S2.2. Combined training objective**

The training process can be marked as:

$$\begin{aligned} \mathcal{L}_{\mathrm{total}}\left( \theta\right)=\mathcal{L}_{\mathrm{quantile}}\left( \theta\right)+\sum_{j\in embed,group,shock} \lambda_{j} C_{j}\left( \theta\right)\quad\left( S-2 \right) \end{aligned}$$

where $\theta$ means all trainable parameters. The first constraint acts on categorical embeddings at the input stage, the second acts on temporal variable selection weights after the embedding has been formed, and the third acts on self-attention after temporal fusion has produced the decoder-side representation.

## **S2.3. Frequency-aware embedding shrinkage**

At the embedding entrance, the risk is that long-tail categories inject high-variance directions, since each category is granted a free embedding row even when evidence for that category is scarce. To counter this, we regulate the energy of each embedding row by the precision of its empirical frequency inside the mini batch. Consider a categorical feature $\mathcal{i}$ with vocabulary size $\mathcal{V}_{\mathcal{i}}$and embedding matrix $W_{i}\in R^{V_{i}\times H}$. Let $c_{i,k}$ be the mini-batch count of the category $k$. For categories observed in the current mini batch, we define the empirical batch frequency $p_{i,k}=c_{i,k}/\sum_{u=1}^{V_{i}} c_{i,u}$. Categories absent from the mini batch are not evaluated in that step. For category $k$ of feature $i$ with batch frequency $p_{i,k}$, define

$$\begin{aligned} R_{\mathrm{embed}}^{\left( i,k \right)}=\frac{\parallel W_{i}\left[ k,: \right]\parallel_{2}^{2}}{\sqrt{p_{i,k}+\varepsilon}}, p_{i,k}=\frac{c_{i,k}}{\sum_{u=1}^{V_{i}} c_{i,u}}\quad\left( S-3 \right) \end{aligned}$$

We impose an $\mathcal{l}_{2}$ penalty on each embedding row that is inversely weighted by the square root of its batch frequency. The factor $1/{\sqrt{P_{i,k}+\varepsilon}}$ reflects the classical $1/\sqrt{n}$ scaling of sampling uncertainty: rarer categories carry higher estimation variance and therefore receive stronger shrinkage. The small constant $\varepsilon> 0$stabilizes the denominator for extremely small but non-zero batch frequencies. Geometrically, the penalty is isotropic in the embedding space, while its strength is category adaptive through $p_{i,k}$, stabilizing long tail rows without unnecessarily constraining frequent categories.

$$\begin{aligned} C_{\mathrm{embed}}=\frac{1}{I}\sum_{i=1}^{I} \left( \frac{1}{V_{i}}\sum_{k=1}^{V_{i}} R_{\mathrm{embed}}^{\left( i,k \right)} \right)\quad\left( S-4 \right) \end{aligned}$$

We first average $R_{\mathrm{embed}}^{\left( i,k \right)}$ across the vocabulary of feature $i$ and then across categorical features. The within-feature factor $1/{\mathcal{V}_{\mathcal{i}}\sum k}$ and across-feature average specify the numerical scaling of the penalty. The coefficient $\lambda_{\text{embed}}$ controls its overall weight. This normalization does not establish invariance to vocabulary composition or equal gradient contributions. Here $I$ denotes the total number of categorical features.

## **S2.4. Historical group-entropy regularization**

After embeddings have been stabilized, the next risk is that many collinear channels diffuse temporal selection weight and confuse subsequent attention. We therefore compress selection at the level of semantic groups that are native to the Temporal Fusion Transformer formulation. Let $w_{t}^{\mathrm{hs}}\in\mathbb{R}^{N_{h}}$ be the nonnegative weights assigned by the historical variable selection at time $\mathcal{t}$ and let $w_{t}^{\mathrm{fut}}\in\mathbb{R}^{N_{f}}$ be the nonnegative weights assigned to future known inputs. We introduce a fixed binary assignment matrix $G\in\{0,1{\}}^{3\times N_{h}}$ with $G_{g,j}=1$ if variable $j$ belongs to a group $g$ $\in\left\{ \text{unknown},\text{known},\text{observed} \right\}.$ Aggregation and normalization are:

$$\begin{aligned} \text{s}_{\text{t}}^{\text{hs}}\text{=}\text{G}\text{w}_{\text{t}}^{\text{hs}}\text{∈}\text{R}^{\text{3}}\text{, }\text{p}_{\text{g}}^{\text{hs}}\left( \text{t} \right)\text{=}\frac{\text{s}_{\text{t}\text{,}\text{g}}^{\text{hs}}}{\sum_{\text{g}\text{'=1}}^{\text{3}} \text{s}_{\text{t}\text{,}\text{g}\text{'}}^{\text{hs}}\text{+}\text{ε}}\quad\left( S-5 \right) \end{aligned}$$

Here, $\varepsilon$ is a small positive numerical-stability constant, not an inverse category count. The archived local group-score implementation uses 10⁻⁹. The left relation aggregates channel scores into three nonnegative groups. The right relation rescales them with an additive constant, so their sum is slightly below one. The following expression is an entropy-form concentration penalty on these epsilon-normalized scores. Only future-known exogenous variables are structurally available in the decoder, the historical-side term supplies the nonconstant grouped-selection regularization:

$$\begin{aligned} C_{\text{group}}=\frac{1}{E}\sum_{t=1}^{E} H\left( p_{t}^{\text{hs}} \right), H\left( p \right)=-\sum_{g=1}^{3} p_{g}\log p_{g}\quad\left( S-6 \right) \end{aligned}$$

The historical group-entropy term encourages concentration of selection weights at each encoder step while retaining channel-level pathways. It does not explicitly regularize temporal differences, so lower entropy alone does not demonstrate stable rankings over time or across training runs.

## **S2.5. Shock-alignment regularization**

We encourage alignment between decoder attention and changes in the learned representation. Let $A^{\left( m \right)}\left( t,\tau\right)$ be the causal self-attention weight from the target time $t$ to past time $\tau\leq t$ for head $\mathcal{m}$. We average the attention heads to define a common attention summary

$$\begin{aligned} \bar{A}\left( t,\tau\right)=\frac{1}{M}\sum_{m=1}^{M} A^{\left( m \right)}\left( t,\tau\right)\quad\left( S-7 \right) \end{aligned}$$

Head averaging defines a summary for the alignment objective. Equal expectations, independent errors, and guaranteed noise cancellation are not assumed. Recent-context mass is calculated by summing causal off-diagonal weights over the local window of width $W$:

$$\begin{aligned} a_{t}=\sum_{k=1}^{W} \bar{A}\left( t,t-k \right)\quad\left( S-8 \right) \end{aligned}$$

We set $W=3$to define a short local context. Under the target-specific resampling grids, this corresponds to a 30-minute recent-context window for vital signs and SF ratio, and a 6-hour recent-context window for laboratory targets. We then aggregate the attention of temporally adjacent positions to obtain the short-term lookback strength, to avoid trivial terms, self-loops of $t$ pointing to $t$ are excluded. The local window is a modeling choice, not a clinically validated shock duration. This geometric projection converts a surface into a time series that can be aligned with a state variability index. The companion signal to which this local mass should respond is a measure of the instantaneous variability of the decoder side representation. Let $v_{t}\in\mathbb{R}^{H}$ denote that representation. A label-free proxy for representation change is the Euclidean first difference of the representation, defined as $s_{t}^{\left( 1 \right)}=\parallel v_{t}-v_{t-1}\parallel_{2}$ where $v_{t}$ means the decoder hidden state at time $t$. First-order differencing summarizes successive latent-state changes through $s_{t}^{\left( 1 \right)}$. This proxy avoids explicit change point labels, integrates cleanly with end-to-end training, and matches the temporal resolution of the local historical-attention mass.

$$\begin{aligned} \tilde{s}_{t}^{\left( 1 \right)}=\frac{s_{t}^{\left( 1 \right)}-\mu_{s}}{\sigma_{s}}, \tilde{a}_{t}=\frac{a_{t}-\mu_{a}}{\sigma_{a}}\quad\left( S-9 \right) \end{aligned}$$

Since $a_{t}$ and $s_{t}^{\left( 1 \right)}$ have different scales and units, naive alignment would be dominated by magnitude instead of temporal co-variation. We apply per-sequence standardization to both. This places both series on a comparable, unit-free scale and ensures that the subsequent loss penalizes discrepancies in relative dynamics rather than absolute size. On the set of decoder indices $\mathcal{D}$ , we then implement soft alignment by minimizing the average squared gap

$$\begin{aligned} C_{\mathrm{shock}}=\frac{1}{\left| \mathcal{D} \right|}\sum_{t\mathcal{\in D}} \left( \tilde{a}_{t}-\tilde{s}_{t}^{\left( 1 \right)} \right)^{2}\quad\left( S-10 \right) \end{aligned}$$

$\mathcal{D}$ denotes the decoder indices used in the alignment loss. The objective links standardized latent-state variation to recent-context attention at those positions. It does not establish that latent changes are true clinical shocks, remove noise by construction, or calibrate the predictive quantiles or downstream AKI probabilities. The first decoder difference requires its preceding representation, and attention and representation indices must refer to the same positions.

## **S2.6. Model configuration**

The embedding, group and shock regularization coefficients were set to 10⁻⁶, 10⁻⁴ and 10⁻³, respectively. Their width is 128, dropout 0.3, batch size 64, gradient clipping 1.0, six attention heads, a two-layer LSTM encoder and decoder, a 300-epoch maximum and patience 10. All targets were optimized with Adam at a learning rate of 1×10⁻⁵. Because the decoder side contains only the known-input group, its group-entropy term is constant, the group regularization therefore acts only on the historical variable-selection weights. Hyperparameters were selected by grid search across seven groups using MIMIC-IV only.

## **S3. Forecast performance and comparator evaluation**

## **S3.1. Forecast error and predictive-quantile calibration**

**Table S3. Forecast error and predictive-quantile calibration across cohorts**

| **Target** | **Cohort** | **MAE** | **MAPE (%)** | **P10 Cov. (Pinball)** | **P90 Cov. (Pinball)** |
| --- | --- | --- | --- | --- | --- |
| Blood Pressure | MIMIC-IV (10-seed) | 5.054 ± 0.347 | 6.668 ± 0.441 | 0.069 (1.378) | 0.935 (1.610) |
| Blood Pressure | MIMIC-III | 6.473 | 8.602 | 0.132 (1.872) | 0.842 (2.085) |
| Blood Pressure | EICU | 4.979 | 6.542 | 0.101 (1.058) | 0.870 (1.289) |
| Heart Rate | MIMIC-IV (10-seed) | 4.119 ± 0.112 | 4.676 ± 0.118 | 0.065 (1.135) | 0.938 (1.295) |
| Heart Rate | MIMIC-III | 6.289 | 7.100 | 0.098 (1.560) | 0.897 (1.813) |
| Heart Rate | EICU | 6.654 | 7.020 | 0.060 (1.629) | 0.890 (1.897) |
| Creatinine | MIMIC-IV (10-seed) | 0.301 ± 0.016 | 15.862 ± 0.611 | 0.068 (0.063) | 0.919 (0.088) |
| Creatinine | MIMIC-III | 0.418 | 22.831 | 0.053 (0.110) | 0.864 (0.107) |
| Creatinine | EICU | 0.340 | 17.092 | 0.045 (0.070) | 0.900 (0.137) |
| SpO2 | MIMIC-IV (10-seed) | 1.041 ± 0.020 | 1.093 ± 0.021 | 0.070 (0.332) | 0.939 (0.228) |
| SpO2 | MIMIC-III | 1.895 | 2.030 | 0.127 (0.811) | 0.918 (0.357) |
| SpO2 | EICU | 2.154 | 2.330 | 0.144 (0.700) | 0.928 (0.407) |
| SF Ratio | MIMIC-IV (10-seed) | 16.529 ± 0.460 | 5.244 ± 0.429 | 0.059 (3.754) | 0.926 (3.831) |
| SF Ratio | MIMIC-III | 20.232 | 6.850 | 0.048 (6.069) | 0.903 (6.977) |
| SF Ratio | EICU | 22.25 | 6.63 | 0.046 (6.421) | 0.919 (9.213) |
| Lactate | MIMIC-IV (10-seed) | 0.346 ± 0.034 | 18.199 ± 0.778 | 0.097 (0.075) | 0.924 (0.158) |
| Lactate | MIMIC-III | 0.756 | 26.030 | 0.092 (0.118) | 0.919 (0.344) |
| Lactate | EICU | 0.606 | 21.450 | 0.085 (0.108) | 0.917 (0.078) |
| Respiratory Rate | MIMIC-IV (10-seed) | 1.720 ± 0.025 | 8.614 ± 0.106 | 0.068 (0.500) | 0.927 (0.534) |
| Respiratory Rate | MIMIC-III | 2.748 | 13.820 | 0.086 (0.744) | 0.883 (0.789) |
| Respiratory Rate | EICU | 2.282 | 11.520 | 0.054 (0.602) | 0.895 (0.674) |
| Temperature | MIMIC-IV (10-seed) | 0.619 ± 0.136 | 1.674 ± 0.369 | 0.114 (0.367) | 0.926 (0.702) |
| Temperature | MIMIC-III | 0.554 | 1.370 | 0.166 (0.245) | 0.869 (0.400) |
| Temperature | EICU | 0.752 | 2.080 | 0.108 (0.429) | 0.739 (0.699) |

*MIMIC-IV results are shown as mean ± SD across ten training-seed runs, on a fixed patient partition. P10 Cov. and P90 Cov. indicate the empirical coverage of the 0.10 and 0.90 predictive quantiles, respectively, pinball losses are shown in parentheses. Coverage closer to the nominal quantile level indicates better calibration*.

## **S3.2. Comparator evaluation protocol**

All comparators were evaluated on the complete MIMIC-IV test windows of the seed-2025 OmniTFT run, with identical patient and window identifiers, preprocessing, scalers and forecast positions, only positions with an observed target value were scored. Historical covariates were adapted to each model's input interface using encoder-period values only. Chronos-2 and TimesFM 3.0 used official pretrained weights without fine-tuning (1,2) and contributed P50 forecasts. UniTS used the original code with a publicly distributed zero-shot checkpoint (3) (a third-party reproduction, patch length and stride 16) without fine-tuning. MTLinear used the official NLinear implementation (4), fitted separately for each target on the MIMIC-IV training and validation partitions (seed 2025, Adam with initial learning rate 0.01, batch size 32, 20 epochs, patience 3, complete-linkage grouping with signed correlation distance 1 − r and threshold 0.5).
For the patient-level tests, each patient's MAE was averaged over that patient's forecast positions, and the comparator-minus-OmniTFT difference was tested with a two-sided paired t test, with Benjamini–Hochberg correction (5) across the 32 comparisons (eight targets × four comparators).

**Table S****4. Patient-level comparisons on the MIMIC-IV test set**

| Target | Comparator | ΔMAE [95% CI] | t (df) | p | BH q |
| --- | --- | --- | --- | --- | --- |
| BP | Chronos-2 | +0.548 [+0.384, +0.712] | +6.577 (203) | 3.98e-10 | 5.540e-10 |
| BP | TimesFM 3.0 | +0.271 [+0.130, +0.412] | +3.795 (203) | 1.95e-04 | 2.229e-04 |
| BP | UniTS | +1.417 [+1.229, +1.605] | +14.881 (203) | 2.36e-34 | 5.805e-34 |
| BP | MTLinear | +0.774 [+0.629, +0.919] | +10.529 (203) | 5.80e-21 | 1.092e-20 |
| HR | Chronos-2 | +4.561 [+4.224, +4.898] | +26.546 (574) | 6.57e-102 | 2.630e-101 |
| HR | TimesFM 3.0 | +4.308 [+4.020, +4.596] | +29.431 (574) | 9.44e-117 | 6.040e-116 |
| HR | UniTS | +5.694 [+5.311, +6.077] | +29.175 (574) | 1.92e-115 | 1.022e-114 |
| HR | MTLinear | +4.183 [+3.883, +4.483] | +27.406 (574) | 2.34e-106 | 1.072e-105 |
| SpO₂ | Chronos-2 | +0.728 [+0.687, +0.769] | +34.790 (593) | 2.43e-145 | 1.941e-144 |
| SpO₂ | TimesFM 3.0 | +0.611 [+0.584, +0.638] | +45.125 (593) | 6.29e-194 | 2.014e-192 |
| SpO₂ | UniTS | +0.836 [+0.789, +0.883] | +35.129 (593) | 5.01e-147 | 5.343e-146 |
| SpO₂ | MTLinear | +0.637 [+0.604, +0.670] | +37.866 (593) | 2.14e-160 | 3.417e-159 |
| RR | Chronos-2 | +2.218 [+2.003, +2.433] | +20.360 (234) | 1.04e-53 | 2.762e-53 |
| RR | TimesFM 3.0 | +2.039 [+1.858, +2.220] | +22.168 (234) | 2.04e-59 | 7.242e-59 |
| RR | UniTS | +2.607 [+2.365, +2.849] | +21.258 (234) | 1.46e-56 | 4.656e-56 |
| RR | MTLinear | +2.016 [+1.828, +2.204] | +21.168 (234) | 2.80e-56 | 8.154e-56 |
| Temp | Chronos-2 | −0.027 [−0.044, −0.010] | −3.176 (298) | 0.00165 | 0.00176 |
| Temp | TimesFM 3.0 | −0.046 [−0.060, −0.032] | −6.574 (298) | 2.19e-10 | 3.190e-10 |
| Temp | UniTS | +0.118 [+0.099, +0.137] | +12.073 (298) | 1.37e-27 | 2.913e-27 |
| Temp | MTLinear | +0.049 [+0.034, +0.064] | +6.619 (298) | 1.68e-10 | 2.681e-10 |
| Lactate | Chronos-2 | +0.487 [+0.352, +0.622] | +7.154 (93) | 1.90e-10 | 2.896e-10 |
| Lactate | TimesFM 3.0 | +0.562 [+0.451, +0.673] | +10.090 (93) | 1.32e-16 | 2.345e-16 |
| Lactate | UniTS | +0.964 [+0.808, +1.120] | +12.298 (93) | 3.30e-21 | 6.610e-21 |
| Lactate | MTLinear | +0.361 [+0.242, +0.480] | +6.035 (93) | 3.22e-08 | 4.123e-08 |
| SF Ratio | Chronos-2 | +9.614 [+6.170, +13.058] | +5.576 (64) | 5.30e-07 | 6.518e-07 |
| SF Ratio | TimesFM 3.0 | +7.182 [+4.184, +10.180] | +4.785 (64) | 1.04e-05 | 1.238e-05 |
| SF Ratio | UniTS | +39.710 [+35.547, +43.873] | +19.057 (64) | 4.47e-28 | 1.021e-27 |
| SF Ratio | MTLinear | +11.247 [+8.075, +14.419] | +7.084 (64) | 1.32e-09 | 1.766e-09 |
| Creatinine | Chronos-2 | +0.047 [−0.023, +0.117] | +1.319 (150) | 0.189 | 0.189 |
| Creatinine | TimesFM 3.0 | +0.113 [+0.053, +0.173] | +3.743 (150) | 2.59e-04 | 2.855e-04 |
| Creatinine | UniTS | +0.086 [+0.005, +0.167] | +2.105 (150) | 0.0369 | 0.0381 |
| Creatinine | MTLinear | +0.271 [+0.208, +0.334] | +8.561 (150) | 1.24e-14 | 2.081e-14 |

*ΔMAE = comparator minus OmniTFT, positive values favor OmniTFT. Units follow Table 2. Intervals are ordinary 95% paired t intervals, with two-sided p values, q uses BH correction over the planned 32 comparisons. Patient counts differ by target, a patient may occur in more than one target. Table S3 reports cohort-level summaries and is not a paired comparison with Table S4.*

# **S4. Component ablation and sampling sensitivity**

All configurations were trained with ten seeds (105, 407, 410, 500, 630, 800, 1991, 2000, 2024 and 2025) on the fixed patient partition. Full-model values are the ten-seed results in Table S3, Vanilla TFT values are taken from Table 2 and were not included in the paired tests. For each target, variant-minus-Full MAE differences across the ten matched seeds were compared with two-sided paired t tests (9 degrees of freedom), with Benjamini–Hochberg correction (5) applied within each target separately to the four ablation and the five sensitivity comparisons. Natural sampling appears in Tables 3 and 4 with the same scores, but its q value can differ between the two correction families. Reference thresholds are listed in Section S2.1.

# **S5. Input availability and missingness sensitivity**

In external validation, each unavailable continuous variable was imputed with its MIMIC-IV training-set median, a population-level fallback that has performed comparably to more complex imputation approaches in clinical prediction models (6) and is consistent with established ICU preprocessing practice (7,8). To examine the sensitivity of forecasts to this setting, we used the frozen respiratory-rate model (12-hour history, 2-hour forecast horizon, 10-minute grid) and the first eligible window per patient in 174 patients, providing 1,538 observed future labels. Under the MIMIC-III and eICU unavailable-item patterns (Table S2), the corresponding values were either replaced by reference-partition medians or means, or removed at the source-event level before temporal aggregation, two additional conditions removed the most frequent reference-partition item (HR) or the three most frequent items (HR, SpO₂ and temperature). Target history, forecast origins, static covariates and outcome labels were held fixed across all conditions. Performance was evaluated as MAE on observed labels, with uncertainty estimated from 2,000 shared patient-bootstrap draws (9) (Table 5).

# **S6. Attention and SHAP analyses**

**Analysis windows.** The explanation analyses (Figures 3E–F and 4F, Supplementary Figures S1–S4, Table S5) used the fixed target-specific deployment checkpoints and frozen preprocessing parameters, without retraining, on the same target-specific temporal grids as the main analysis. Each patient contributed one eligible history window, patient counts are given in Table S5.

**Variable selection and temporal attention.** Variable-selection profiles were averaged over historical positions and patients. Temporal attention was taken from the first forecast query, averaged across the six attention heads and normalized over historical positions. Figure 3E shows subgroup differences relative to uniform attention, and Figure 3F shows the attention mass in four consecutive quarters of the history window. Intervals are percentiles from 2,000 patient-bootstrap resamples.

**Grouped SHAP.** Permutation Shapley values (10) attributed the first-step P50 forecast, in target units, to the complete historical trajectory of each clinical input, with reference values sampled from the same clinical item in the reference partition (the MIMIC-IV training set). Sensitivity to the estimation procedure was assessed across ten algorithm seeds and ten background draws, and sampling stability across 2,000 patient-bootstrap resamples, summarized by Spearman rank correlation and Top-10 Jaccard overlap (Table S5, Figure S3). Displayed features were restricted to the clinical variables listed in Table S2. Figure S2 shows up to three features per target with at least 20 patient windows with observed values, no creatinine feature met this criterion.

**Table S5. Patient-sampling stability of variable selection and grouped SHAP, with SHAP algorithm/background sensitivity**

| Analysis | Target | Patients / bootstrap draws | Spearman ρ | Top-10 Jaccard | SHAP retention (%) Patient / Algorithm / Background |
| --- | --- | --- | --- | --- | --- |
| Variable selection | BP | 76 patients, one window each 2,000 draws | 0.970 [0.954, 0.981] | 0.818 [0.538, 1.000] | — |
| Variable selection | HR | 474 patients, one window each 2,000 draws | 0.985 [0.979, 0.990] | 0.667 [0.538, 1.000] | — |
| Variable selection | SpO2 | 488 patients, one window each 2,000 draws | 0.985 [0.978, 0.990] | 0.818 [0.538, 1.000] | — |
| Variable selection | RR | 174 patients, one window each 2,000 draws | 0.979 [0.968, 0.986] | 0.667 [0.538, 1.000] | — |
| Variable selection | Temperature | 194 patients, one window each 2,000 draws | 0.982 [0.973, 0.988] | 1.000 [0.818, 1.000] | — |
| Variable selection | Creatinine | 79 patients, one window each 2,000 draws | 0.933 [0.874, 0.968] | 0.667 [0.429, 1.000] | — |
| Variable selection | Lactate | 69 patients, one window each 2,000 draws | 0.909 [0.855, 0.945] | 0.667 [0.538, 1.000] | — |
| Variable selection | SF | 35 patients, one window each 2,000 draws | 0.941 [0.907, 0.965] | 0.818 [0.818, 1.000] | — |
| Temporal attention | BP | 76 patients, one window each 2,000 draws | 0.793 [0.644, 0.879] | — | — |
| Temporal attention | HR | 474 patients, one window each 2,000 draws | 0.884 [0.803, 0.936] | — | — |
| Temporal attention | SpO2 | 488 patients, one window each 2,000 draws | 0.868 [0.782, 0.927] | — | — |
| Temporal attention | RR | 174 patients, one window each 2,000 draws | 0.750 [0.606, 0.846] | — | — |
| Temporal attention | Temperature | 194 patients, one window each 2,000 draws | 0.792 [0.666, 0.878] | — | — |
| Temporal attention | Creatinine | 79 patients, one window each 2,000 draws | 0.867 [0.510, 0.972] | — | — |
| Temporal attention | Lactate | 69 patients, one window each 2,000 draws | 0.944 [0.846, 0.993] | — | — |
| Temporal attention | SF | 35 patients, one window each 2,000 draws | 0.878 [0.763, 0.937] | — | — |
| Grouped SHAP | BP | 76 patients, one window each 2,000 draws | 0.927 [0.854, 0.967] | 0.667 [0.538, 1.000] | 83.49 / 80.00 / 81.00 |
| Grouped SHAP | HR | 474 patients, one window each 2,000 draws | 0.976 [0.962, 0.985] | 0.818 [0.667, 1.000] | 91.24 / 93.00 / 96.00 |
| Grouped SHAP | SpO2 | 488 patients, one window each 2,000 draws | 0.973 [0.948, 0.985] | 0.818 [0.667, 1.000] | 89.48 / 90.00 / 89.00 |
| Grouped SHAP | RR | 174 patients, one window each 2,000 draws | 0.946 [0.904, 0.980] | 0.818 [0.667, 1.000] | 92.18 / 91.00 / 88.00 |
| Grouped SHAP | Temperature | 194 patients, one window each 2,000 draws | 0.938 [0.874, 0.983] | 0.818 [0.538, 1.000] | 88.16 / 90.00 / 84.00 |
| Grouped SHAP | Creatinine | 79 patients, one window each 2,000 draws | 0.861 [0.686, 0.974] | 0.818 [0.538, 1.000] | 86.91 / 88.00 / 92.00 |
| Grouped SHAP | Lactate | 69 patients, one window each 2,000 draws | 0.865 [0.750, 0.954] | 0.667 [0.538, 0.818] | 80.95 / 87.00 / 83.00 |
| Grouped SHAP | SF | 35 patients, one window each 2,000 draws | 0.940 [0.869, 0.975] | 0.818 [0.538, 1.000] | 85.85 / 97.00 / 89.00 |

*Patient-bootstrap rows report the median and 2.5th–97.5th percentiles of agreement with the full-sample profile across 2,000 draws. Each patient contributes one intact window. The retention column gives the mean percentage of fixed full-sample Top-10 SHAP features retained under patient resampling, ten algorithm seeds and ten background draws, in that order.*

**Subgroups.** Subgroups were defined by sex, age at admission (<65 versus ≥65 years) (11), and observed-input coverage (lower versus higher, split at the median of the reference partition). In grouped-SHAP subgroup contrasts (Figure S4), subgroup intervals were obtained from 2,000 within-group patient-bootstrap resamples. These analyses describe the fitted models and do not establish causal or physiological relationships (12).

# **S7. Forecast-augmented AKI analysis**

Incident AKI was defined by the KDIGO creatinine criteria (13). Except for the per-setting AUROC intervals in Table 6, which are DeLong 95% CIs, uncertainty for Table 6, Figure 5I and Figure S5 was estimated from 2,000 paired hospital-cluster bootstrap resamples (9) of the fixed out-of-fold probabilities, without refitting. AP was computed without interpolation (14). Calibration-in-the-large was estimated with the predicted log odds as an offset, and the calibration slope with a free intercept. In Figure 5H, Forecast 48h is the last forecast median, Forecast change the final minus first forecast median, the P90–P10 span max(P90) − min(P10) across the horizon, and draws the number of observed creatinine measurements, including pre-ICU values.

**Table S6. Top five features of SHAP**

| **Feature** | **Rank** | **Variable** |
| --- | --- | --- |
| Blood Pressure | 1 | Creatine Kinase (CK) |
|  | 2 | Ferritin |
|  | 3 | Aspartate Aminotransferase (AST) |
|  | 4 | Iron Binding Capacity, Total |
|  | 5 | Triglycerides |
| Heart Rate | 1 | Creatine Kinase (CK) |
|  | 2 | Hemoglobin |
|  | 3 | Lactate Dehydrogenase (LD) |
|  | 4 | NTproBNP |
|  | 5 | Ferritin |
| Creatinine | 1 | Ferritin |
|  | 2 | Urea Nitrogen, Urine |
|  | 3 | Creatine Kinase (CK) |
|  | 4 | Lactate Dehydrogenase (LD) |
|  | 5 | PEEP |
| SpO₂ | 1 | Lactate Dehydrogenase (LD) |
|  | 2 | Ferritin |
|  | 3 | RBC |
|  | 4 | Creatine Kinase (CK) |
|  | 5 | Osmolality, Urine |
| SF ratio | 1 | Bilirubin, Direct |
|  | 2 | Tidal Volume |
|  | 3 | Alveolar-arterial Gradient |
|  | 4 | Urea Nitrogen, Urine |
|  | 5 | Lactate Dehydrogenase (LD) |
| Lactate | 1 | Alveolar-arterial Gradient |
|  | 2 | Lactate Dehydrogenase (LD) |
|  | 3 | Tidal Volume |
|  | 4 | Alanine Aminotransferase (ALT) |
|  | 5 | Potassium |
| Respiratory Rate | 1 | Ferritin |
|  | 2 | Creatine Kinase (CK) |
|  | 3 | Hematocrit |
|  | 4 | Aspartate Aminotransferase (AST) |
|  | 5 | Lactate Dehydrogenase (LD) |
| Temperature | 1 | Creatine Kinase (CK) |
|  | 2 | Lactate Dehydrogenase (LD) |
|  | 3 | Protein |
|  | 4 | Osmolality, Measured |
|  | 5 | Respiratory Rate (spontaneous) |

**S8. Computational cost and deployment**

Frozen prediction calls were measured with TensorFlow 2.15.0 on an NVIDIA H100 80GB environment, processing batches of up to 128 windows. The eight target-specific checkpoints contain about 1.8M parameters each. Each complete target benchmark workload contained 35–488 windows. The total warm prediction time over a complete target workload was 0.129–0.709 s, and peak allocated GPU memory about 80MiB. These measurements exclude data extraction, harmonization and output writing. A complete OmniTFT training run required approximately 4–6 h per seed on a single NVIDIA H100 GPU.

The contemporary comparators were run on NVIDIA H100 GPUs. Chronos-2, TimesFM 3.0 and UniTS remained frozen, their summed timed prediction calls were 62.07, 74.50 and 1.40 minutes, respectively. Chronos-2 used batches of 32 followed by 8 for BP, HR and SpO2, and batches of 8 for the other targets, TimesFM used 32 and UniTS 128. MTLinear used eight 20-epoch fits with training batches of 32 and validation batches of 1,024. Its cumulative optimization, validation and target-prediction times were 88.56 minutes, 18.48 seconds and 0.293 seconds, respectively. All eight fits completed the 20-epoch budget, retaining validation-selected parameters.

Each target instance requires its own checkpoint, scaler, input dictionary and supported history and forecast horizon. Practical deployment requires patient/admission isolation, unit and range checks, monitoring of input and prediction availability, version control, and local assessment of forecast calibration and any alert threshold. Forecasted measurements must be distinguishable from observations.

References

1.	Ansari AF, Shchur O, Küken J, Auer A, Han B, Mercado P, Rangapuram SS, Shen H, Stella L, Zhang X, et al. Chronos-2: From Univariate to Universal Forecasting. (2025) doi: 10.48550/arXiv.2510.15821

2.	Jain A, Sen R. TimesFM-3: A zero-shot foundation model for multivariate forecasting. *Google Research* (2026) https://research.google/blog/timesfm-3-a-zero-shot-foundation-model-for-multivariate-forecasting/ [Accessed September 17, 2026]

3.	Gao S, Koker T, Queen O, Hartvigsen T, Tsiligkaridis T, Zitnik M. UniTS: A Unified Multi-Task Time Series Model. *Advances in Neural Information Processing Systems*. Curran Associates, Inc. (2024). p. 140589–140631 doi: 10.52202/079017-4463

4.	Nochumsohn L, Zisling H, Azencot O. A Multi-Task Learning Approach to Linear Multivariate Forecasting. *Proceedings of The 28th International Conference on Artificial Intelligence and Statistics*. Proceedings of Machine Learning Research. PMLR (2025). p. 2638–2646 https://proceedings.mlr.press/v258/nochumsohn25a.html

5.	Benjamini Y, Hochberg Y. Controlling the False Discovery Rate: A Practical and Powerful Approach to Multiple Testing. *Journal of the Royal Statistical Society: Series B (Methodological)* (1995) 57:289–300. doi: 10.1111/j.2517-6161.1995.tb02031.x

6.	Berkelmans GFN, Read SH, Gudbjörnsdottir S, Wild SH, Franzen S, van der Graaf Y, Eliasson B, Visseren FLJ, Paynter NP, Dorresteijn JAN. Population median imputation was noninferior to complex approaches for imputing missing values in cardiovascular prediction models in clinical practice. *Journal of Clinical Epidemiology* (2022) 145:70–80. doi: 10.1016/j.jclinepi.2022.01.011

7.	Wang S, McDermott MBA, Chauhan G, Ghassemi M, Hughes MC, Naumann T. MIMIC-Extract: A Data Extraction, Preprocessing, and Representation Pipeline for MIMIC-III. *Proceedings of the ACM Conference on Health, Inference, and Learning*. (2020). p. 222–235 doi: 10.1145/3368555.3384469

8.	Hoogland J, van Barreveld M, Debray TPA, Reitsma JB, Verstraelen TE, Dijkgraaf MGW, Zwinderman AH. Handling missing predictor values when validating and applying a prediction model to new patients. *Statistics in Medicine* (2020) 39:3591–3607. doi: 10.1002/sim.8682

9.	Field CA, Welsh AH. Bootstrapping clustered data. *Journal of the Royal Statistical Society: Series B (Statistical Methodology)* (2007) 69:369–390. doi: 10.1111/j.1467-9868.2007.00593.x

10.	Lundberg SM, Lee S-I. A Unified Approach to Interpreting Model Predictions. *Advances in Neural Information Processing Systems*. (2017). p. 4765–4774 https://proceedings.neurips.cc/paper/2017/hash/8a20a8621978632d76c43dfd28b67767-Abstract.html

11.	Docherty AB, Anderson NH, Walsh TS, Lone NI. Equity of Access to Critical Care Among Elderly Patients in Scotland: A National Cohort Study. *Critical Care Medicine* (2016) 44:3–13. doi: 10.1097/CCM.0000000000001377

12.	Jain S, Wallace BC. Attention is not Explanation. *Proceedings of the 2019 Conference of the North American Chapter of the Association for Computational Linguistics: Human Language Technologies, Volume 1 (Long and Short Papers)*. (2019). p. 3543–3556 doi: 10.18653/v1/N19-1357

13.	Khwaja A. KDIGO Clinical Practice Guidelines for Acute Kidney Injury. *Nephron Clinical Practice* (2012) 120:c179–c184. doi: 10.1159/000339789

14.	Saito T, Rehmsmeier M. The Precision-Recall Plot Is More Informative than the ROC Plot When Evaluating Binary Classifiers on Imbalanced Datasets. *PLOS ONE* (2015) 10:e0118432. doi: 10.1371/journal.pone.0118432

## Supplementary Figure S1. Eight-target patient-window grouped SHAP distributions.

Each target displays the ten eligible clinical variables with highest mean absolute grouped attribution within the matched original-variable inventory. Each point is one patient window’s signed contribution of that item’s historical input trajectory to first forecast-step P50, in target units. Target history and other players remain in the full attribution game but are omitted from this display. Exact zeros and extrema are retained. Color indicates the within-feature percentile of the observed historical mean, grey indicates no retained observed value. Symbols denote <20 observed windows (†), one (!) or none (‡). Patient counts, window geometry and estimator settings are given in Section S6 and Table S5. Unlike Figure 4B, which shows event-level SHAP values, these panels show grouped patient-window attributions.

## Supplementary Figure S2. Supported historical-value and grouped-attribution associations.

Each of seven targets contributes three supported measurement features selected by overall mean absolute attribution within the original-variable subset. Eligibility requires ≥20 patient windows with retained observed values and ≥2 distinct historical means, creatinine has no qualifying feature (maximum support 17). Each point pairs a patient window’s observed historical mean with the grouped contribution of the complete item trajectory to first-step P50. Values, zero attributions and extrema are retained without fitted trends or significance tests. Units in brackets follow the source dictionary or original variable inventory (Table S2), without additional unit conversion.

## Supplementary Figure S3. Grouped-attribution stability and patient-influence diagnostics.

All panels use the same eligible subset and fixed overall Top-10 as Figure S1. (A) Median Spearman correlation and positive-only Top-10 Jaccard overlap across 2,000 patient-bootstrap profiles, intervals span the 2.5th–97.5th percentiles. (B) Mean inclusion of the fixed ten features under patient resampling, ten algorithm seeds at one background and ten background draws at one algorithm seed. (C) Remaining mean absolute attribution after each feature’s largest contributor is omitted, as a percentage of its full-sample value, different features may omit different patients. (D) Ranks of the original ten features after omitting one maximum-total-attribution patient per target and reranking all eligible inputs. Ties are ordered by feature identifier. All 80 cells remain, arrows indicate direction and 16 ranks change. These diagnostics use the stored attributions: no patient is removed from the primary analysis, and no model prediction or SHAP value is re-estimated.

## Supplementary Figure S4. Fixed-feature grouped-attribution contrasts across subgroups.

Eight target rows and three columns compare female minus male, age <65 minus ≥65 years and lower minus higher observed-item coverage. Each panel retains the same ten features and order as Figure S1. Points are differences in mean absolute grouped attribution, in target units, bars are percentile 95% intervals from 2,000 independent within-group patient-bootstrap draws. Coverage was defined using all model input items and split at the MIMIC-IV training-set median, with ties assigned to the higher group. Symbols denote whole-cohort observed support <20 (†), one (!) or none (‡), open points flag fewer than two observed patients in either subgroup. All features and full interval ranges remain, including sparse and zero-spanning results. Group sizes and definitions are in Section S6.

## Supplementary Figure S5. Uncertainty and complementary assessments of the AKI analysis.

Analyses use the fixed out-of-fold risks for 4,685 stays from 178 hospitals: 45 stage ≥2 events and 374 stage ≥1 events. Points and bars show estimates and 95% percentile intervals from 2,000 paired hospital-bootstrap draws. (A) Stage ≥2 AUROC and AP. (B) Calibration-in-the-large with the predicted-log-odds coefficient fixed at one, and calibration slope with a free intercept, ideal values are zero and one. These diagnostics do not recalibrate risks. (C) OmniTFT-minus-comparator net benefit per 1,000 stays at thresholds of 0.5%, 1%, 2% and 3%, intervals are pointwise. (D) Precision at 10%, 20% and 30% alert burdens, complementing Figure 5F, pooled out-of-fold thresholds remain fixed during resampling. (E) Secondary stage ≥1 paired differences in AUROC, AP and Brier, Brier differences are multiplied by 1,000 and negative values favor OmniTFT. (F) Stage ≥2 AUROC increments versus Real-24h for single-measurement histories (909 stays, 17 events), multiple-measurement histories (3,776 stays, 28 events), and the single-minus-multiple interaction. Counts include pre-ICU creatinine records, the interaction interval spans zero. The fixed-risk intervals exclude refitting uncertainty.
